# Oleic acid as potential immunostimulant in metabolism pathways of hybrid grouper fingerlings (*Epinephelus fuscoguttatus *× *Epinephelus lanceolatus*) infected with *Vibrio vulnificus*

**DOI:** 10.1038/s41598-023-40096-7

**Published:** 2023-08-08

**Authors:** Maya Erna Natnan, Chen-Fei Low, Chou-Min Chong, Hamidun Bunawan, Syarul Nataqain Baharum

**Affiliations:** 1 Metabolomics Research Laboratory, Institute of Systems Biology (INBIOSIS), Universiti Kebangsaan Malaysia, UKM, 43600 Bangi, Selangor Malaysia; 2 Laboratory of Immunogenomics, Department of Aquaculture, Faculty of Agriculture, Universiti Putra Malaysia, 43400 Serdang, Selangor Malaysia

**Keywords:** Metabolomics, Metabolic engineering

## Abstract

Grouper culture has been expanding in Malaysia due to the huge demand locally and globally. However, due to infectious diseases such as vibriosis, the fish mortality rate increased, which has affected the production of grouper. Therefore, this study focuses on the metabolic profiling of surviving infected grouper fed with different formulations of fatty acid diets that acted as immunostimulants for the fish to achieve desirable growth and health performance. After a six-week feeding trial and one-week post-bacterial challenge, the surviving infected grouper was sampled for GC–MS analysis. For metabolite extraction, a methanol/chloroform/water (2:2:1.8) extraction method was applied to the immune organs (spleen and liver) of surviving infected grouper. The distribution patterns of metabolites between experimental groups were then analyzed using a metabolomics platform. A total of 50 and 81 metabolites were putatively identified from the spleen and liver samples, respectively. Our further analysis identified glycine, serine, and threonine metabolism, and alanine, aspartate and glutamate metabolism had the most impacted pathways, respectively, in spleen and liver samples from surviving infected grouper. The metabolites that were highly abundant in the spleen found in these pathways were glycine (20.9%), l-threonine (1.0%) and l-serine (0.8%). Meanwhile, in the liver l-glutamine (1.8%) and aspartic acid (0.6%) were found to be highly abundant. Interestingly, among the fish diet groups, grouper fed with oleic acid diet produced more metabolites with a higher percent area compared to the control diets. The results obtained from this study elucidate the use of oleic acid as an immunostimulant in fish feed formulation affects more various immune-related metabolites than other formulated feed diets for vibriosis infected grouper.

## Introduction

Groupers are among the popular tropical marine finfish that have been widely cultured and captured not only in Malaysia but also in other Asian-Pacific region countries such as Taiwan, Indonesia, China, and Japan^1^. In Malaysia, hybrid grouper (*E. fuscoguttatus* female × *E. lanceolatus* male) was first produced at the Borneo Marine Research Institute of Universiti Malaysia Sabah^2^. Since then, the hybrid grouper has become the fast-growing marine finfish in the South-East Asian region^3^. To improve productivity and fulfil the market demand, groupers are cultured intensively in many fish farms. However, intensive mariculture practices such as high stocking density had caused negative effects on regards to their growth performance and grouper susceptibility to infectious diseases^4^.

Vibriosis has been considered one of the most common diseases that causes a serious economic loss in the wide range of cultured marine fish species^4,5^. In previous studies, it has been reported that the main mortalities in several grouper aquaculture farms were caused by vibriosis infection^6–11^. According to a previous report, vibriosis infection had been widespread within the cultured grouper in Malaysia, where the presence of *Vibrio* spp. including *Vibrio communis* (28%), *Vibrio parahaemolyticus* (25%), *Vibrio alginolyticus* (19%) and *Vibrio vulnificus* (14%) were majorly detected in grouper farms^10^. In another study, an outbreak of vibriosis had caused more than 50% mortality of grouper cultured, in the deep-sea cages in Langkawi. From the report, two main *Vibrio* spp. were identified including *V. vulnificus* and *V. alginolyticus*^12^*.* After infection with *Vibrio*, fish usually will develop several symptoms including skin discolouring, external hemorrhage, gill necrosis, lesion on the skin, hemorrhagic liver, and lastly lethality^13,14^. Aside from infections of aquatic animals, *Vibrio* spp. also responsible for the contaminated food, especially seafood. *Vibrio vulnificus, V. parahaemolyticus*, and *Vibrio cholerae* are among the common foodborne infections occurred in humans^15^.

Generally, current approaches such as antibiotics had been used for controlling and preventing pathogens infection. Nevertheless, the emergence of antibiotic-resistant bacteria strain poses a serious challenge to antibiotics efficiency^16^. In another study, some antibiotics can cause oxidative stress effects in fish, for example, the florfenicol that elevated the superoxide dismutase (SOD), catalase (CAT), and malondialdehyde (MDA) of European seabass (*Dicentrarchus labrax*)^17^. The aggregation and imbalance of these free radicals could cause cells and tissues damage^18^. For this reason, it is imperative to find new alternatives that provide good nutrition to cultured aquatic animals while ensuring food quality, safety, and environmental sustainability. Since antibiotics showed some restrictions used in aquaculture, the development of additives such as amino acids, organic acids, and fatty acids as an immunostimulant gave new insight and strategy in controlling infectious diseases in fish culture^19^. According to Sankian et al.^20^, a feeding diet experiment conducted on mandarin fish, *Siniperca scherzeri* with soybean oil and linseed oil comprising fatty acid components such as oleic acid, linoleic acid, and α-linoleic acid demonstrated remarkable improvement in their survival and growth performance. From our previous study, key metabolites from the fatty acid group namely oleic acid, stearic acid, palmitic acid, behenic acid, palmitoleic acid, cis-erucic acid, and 8,11-eicosadienoic acid, are abundant in grouper that survived the *V. vulnificus* infection^21^. This finding is consistent with the study by Natnan et al.^22^, who showed that the exogenous of oleic acid, palmitic acid, stearic acid and behenic acid in the feed formulation can increase the immune response while simultaneously helping to improve survival^22^.

Past reviews in omics strategies contribute to improving numerous aspects of the aquaculture value chain by understanding not only the fish genetic variations, but also the key metabolites and protein expression changes that are involved in immune response toward infectious fish disease^23^. The reviews outlined that the use of omics application provided important contributions to the future improvement of fish farmed productivity and quality^24,25^. In this study, a metabolomic approach using the GC–MS-based metabolomics techniques was selected because it generates reproducible molecular fragment patterns with high chromatographic resolution and robust a database from identified peaks, which directly help provide information on fish metabolic responses and pathways to inform further discoveries of fish physical and biochemical properties^26^, particularly in response to vibriosis disease. This study also attempted to characterize and target metabolites correspond to the surviving infected grouper fed with four different potential of fatty acid immunostimulants.

## Result

### Survival rate of grouper infected with *V. vulnificus*

**Figure 1:**
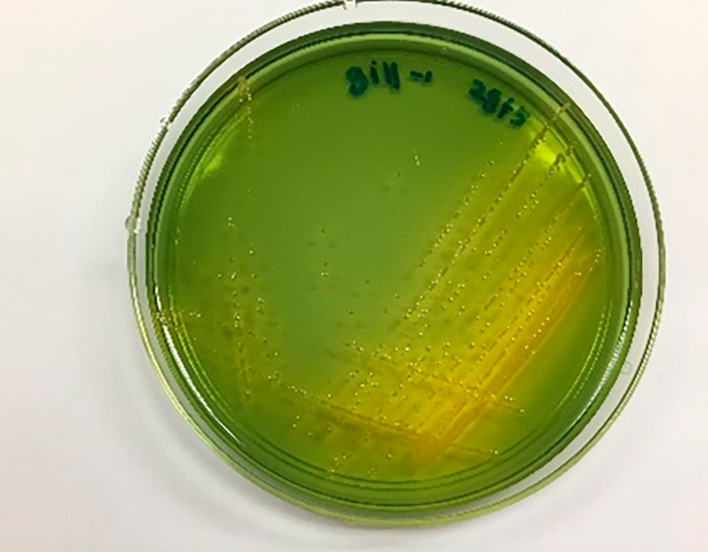
*Vibrio* grow on the TCBS agar plate swabbed from the fish gill.

After six weeks of feeding trial and one week of the post-bacterial challenge, the survival rate of grouper in each feeding group was calculated. There have a significant percentage of oleic acid diet (63.3%), stearic acid diet (53.3%), palmitic acid diet (53.3%), behenic acid diet (50.0%) and control diet (43.3%). Figure 1 shows the colonies of *Vibrio* sp. grew on the TCBS agar plate. The yellowish colonies indicated the presence of *Vibrio* sp. bacteria isolated from the fish samples infected with *V. vulnificus*.

### Metabolites profiling of groupers fed with different feed formulation

**Figure 2:**
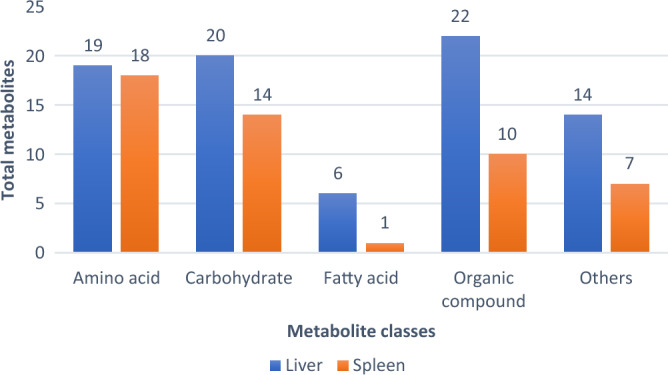
Comparison of the total identified metabolites between liver and spleen.

The GCMS-based metabolomics analysis was conducted on the liver and spleen samples of the survived-infected grouper. Figure 2 presents the total metabolites identified in the liver and spleen. A total of 91 metabolites were identified in both liver and the spleen samples of the infected grouper, which belong to several major classes including amino acid (25.3%), carbohydrate (22.0%), fatty acid (6.6%), and an organic compound (27.5%). Moreover, other metabolites (18.7%) were classified into aldehyde, alkane, nitrile, inorganic compounds, hydrocarbon, siloxane, pyrimidine, and monocarboxylic acid. In the liver, 81 metabolites were identified compared to only 50 metabolites identified in the spleen.

**Figure 3:**
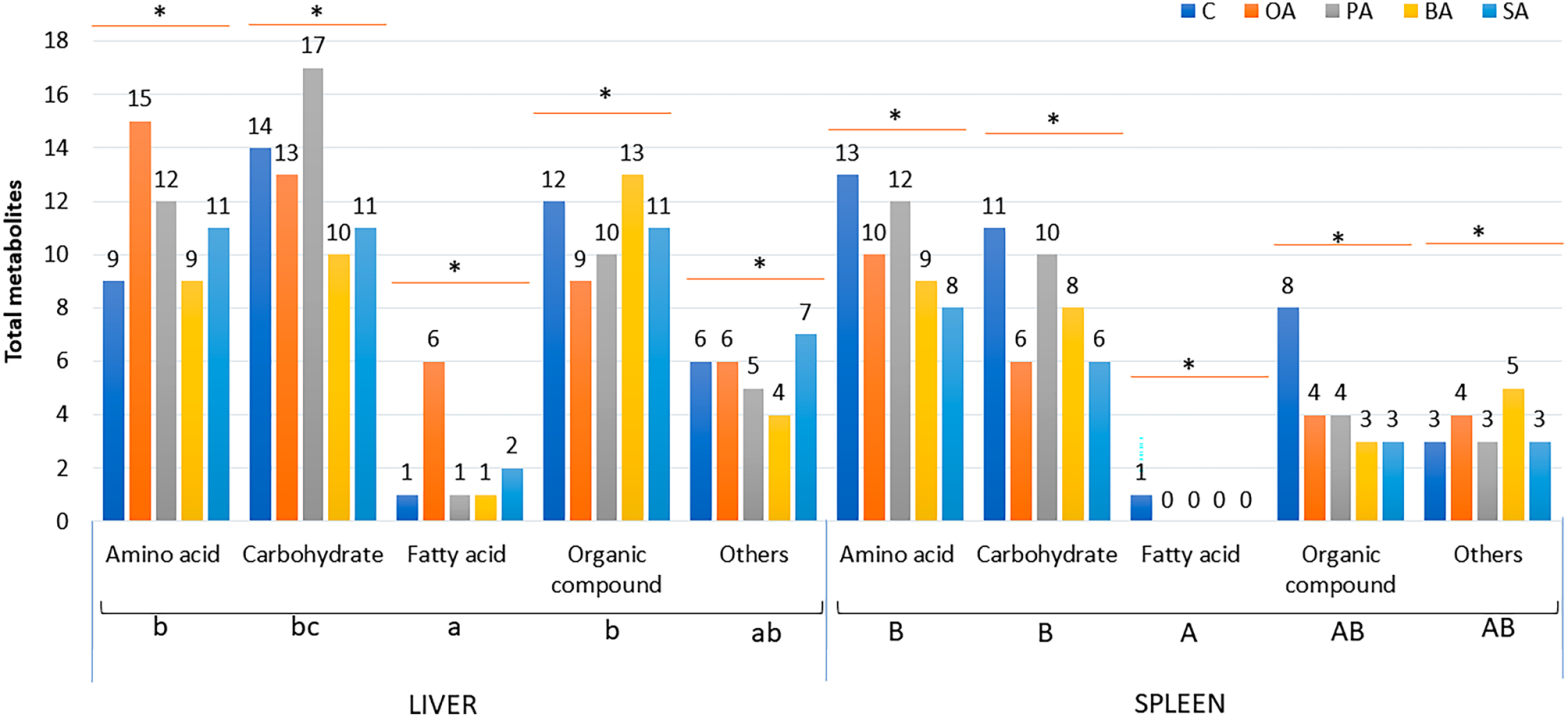
Comparison of the total identified metabolites in the liver and spleen sample of challenged grouper in corresponded to different feed formulation C; fed with the control diet, OA; fed with the oleic acid diet, PA; fed with the palmitic acid diet, BA; fed with the behenic acid diet and SA; fed with the stearic acid diet. Different letters indicate significant different (*p* < 0.05) and * indicated no significant different between diet groups (*p* > 0.05).

Figure 3 shows the comparison of the total metabolites present in the liver and the spleen of survived-infected grouper fed with five different formulated diets. From the result the highest number of metabolites identified in the liver was for grouper fed with the oleic acid (49 metabolites) formulation diet, followed by other fatty acid formulated diet groups including palmitic acid; 45 metabolites, behenic acid; 37 metabolites, stearic acid; 42 metabolites, and control; 42 metabolites. Meanwhile, in the spleen, the highest number of metabolites identified was for grouper fed with the control diet (39 metabolites) followed by the palmitic acid diet (29 metabolites), behenic acid diet (25 metabolites), oleic acid diet (24 metabolites) and stearic acid diet (20 metabolites).

Two major classes comprised of amino acids, and carbohydrates showed the most metabolites detected in the liver and the spleen of all feeding treatment groups indicating fish produced important metabolites in response to the *Vibrio* infection. In the control diet, higher carbohydrate metabolites were detected in the liver (14) samples compared with spleen (11) samples. The total number of amino acid metabolites in the liver and spleen was 9 and 13, respectively. Fatty acid metabolites had the lowest metabolite detection, with one metabolite detected in the liver and spleen of the control diet group. For groupers fed oleic acid, amino acid metabolites were detected the highest in the liver (15), and in the spleen (10), it is the third highest metabolite detected when compared to other feed diet groups. The grouper feeding on oleic acid also exhibited many other metabolites in the liver, including carbohydrates (13), fatty acids (6) and organic acids (9). Meanwhile in the spleen, lesser metabolites were detected including carbohydrates (6), and organic acids (4). For other dietary formulations, including palmitic acid, behenic acid, and stearic acid, metabolites were detected in similar amounts, with higher amounts detected in liver samples compared to spleen samples (Fig. 3).

Non-parametric Kruskal–Wallis’s test was performed for this not normally distributed data using the statistical software program SPSS 25.0. The analysis demonstrated that the five formulated diets had no significant effect (*p* > *0.05*) on the same group of metabolites. However, when analysing among different group of compounds, their total metabolites showed significance different (*p* < *0.05*).

A list of all the identified metabolites were shown in Supplementary Table 1. Following are the percent area of metabolites in both liver and spleen samples. In the liver, carbohydrate compounds including d-galactose (ranging from 41.5% to 58.2%) was produced at the higher levels followed by d-glucose (ranging from 7.5% to 11.0%), thymol-α-d-glucopyranoside (ranging from 4.2% to 10.9%), d-mannose (ranging from 1.8% to 7.9%) and d-ribose (ranging from 1.3% to 1.9%). Similar metabolites were also found in the spleen, where d-galactose (ranging from 21.0% to 45.9%) was produced at the higher levels followed by d-glucose (ranging from 7.0% to 10.5%), thymol-α-d-glucopyranoside (ranging from 5.3% to 8.3%), d-mannose (ranging from 1.0% to 4.7%) and d-ribose (ranging from 2.2% to 3.6%).

Regarding the amino acid compounds, glycine (ranging from 7.9% to 13.7%) was shown to have the highest-level percent area in the liver samples compared to other diet groups (Supplementary Table 1). It was followed by l-valine (1.9%), l-glutamine (ranging from 0.4% to 1.8%), N-α-acetyl-L-lysine (ranging from 0.4% to 1.7%), l-threonine (ranging from 0.2% to 0.9%), l-leucine (0.1% to 0.7%), l-serine (ranging from 0.2% to 0.4%) and l-isoleucine (ranging from 0.1% to 0.3%). Similarly, in the spleen, glycine (ranging from 14.2% to 20.9%) have the highest-level percent area, followed by l-valine (0.1% to 12.31%), l-glutamine (ranging from 1.6% to 6.4%), l-aspartic acid (ranging from 2.2% to 3.6%), l-alanine (ranging from 1.8% to 3.2%), pyroglutamic acid (1.2%), l-threonine (0.4% to 1.0%) and l-serine (0.3% to 0.8%). Oleic acid metabolites (1.49%) only can be found in liver samples of grouper fed dietary oleic acid. Majority of fatty acids also can be found in grouper fed dietary oleic acid including. octadecanoic acid (0.2%), hexadecanoic acid (3.17%), and 9,12-octadecadienoic acid (0.4%). (Supplementary Table 1).

### Metabolites comparison between liver and spleen

**Figure 4:**
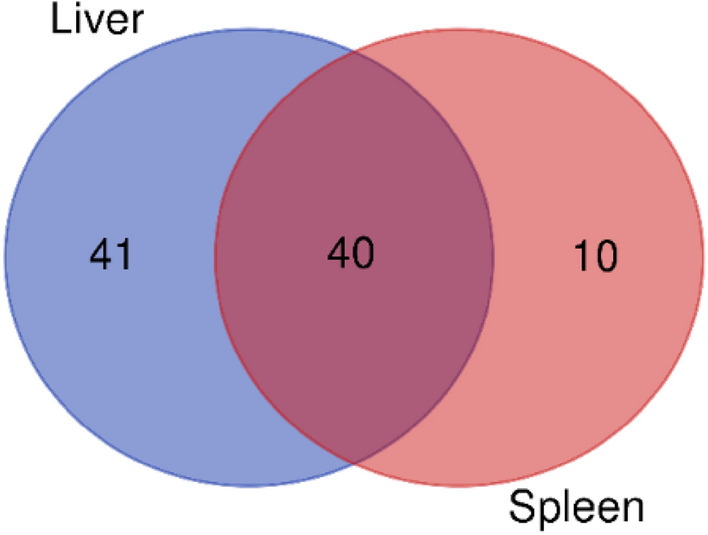
Venn diagram of the overall distribution of metabolites in the liver and the spleen of survived-infected grouper in all feeding treatments.

The distribution of the significantly different metabolites is shown below in the Venn diagram. Based on Fig. 4, a higher number of unique metabolites were identified in the liver, with a total of 41 metabolites compared to 10 metabolites identified in the spleen of the survived-infected grouper. It is also observed that there are 40 metabolites present in both the liver and spleen of survived-infected grouper.

**Figure 5:**
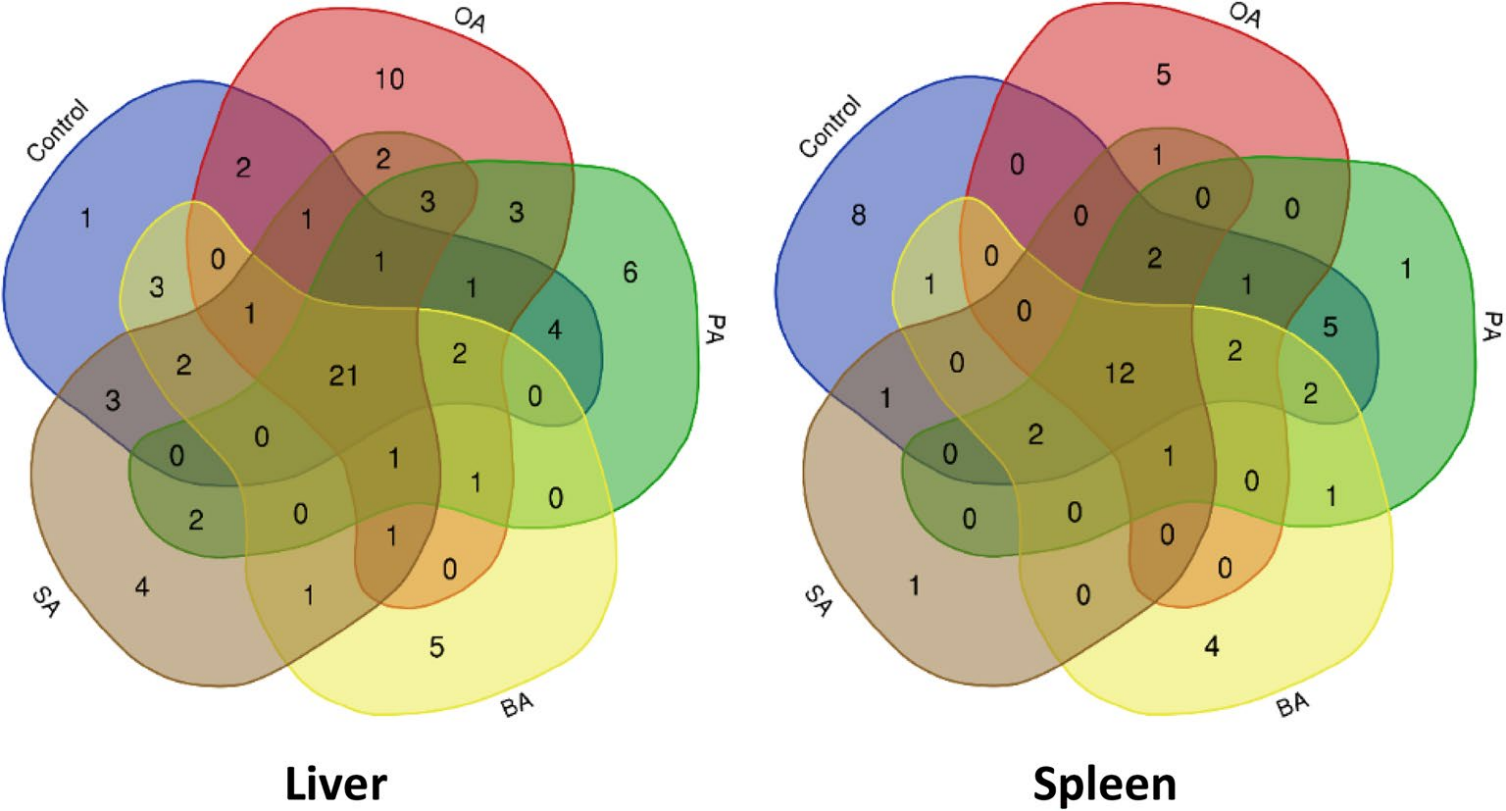
Venn diagram represent the similarities and differences of identified metabolites obtained from two immune organs (liver and spleen) of infected hybrid grouper fed with five different formulated diet. C; infected and fed with the control diet, OA; infected and fed with oleic acid, PA; infected and fed with palmitic acid, BA; infected and fed with behenic acid and SA; infected and fed with stearic acid.

Meanwhile, the Venn diagram shown in Fig. 5 demonstrated more comprehensive similarities and differences between metabolites in the liver and the spleen of survived-infected hybrid grouper for five different formulated diets. According to Fig. 5, a total of 21 metabolites were successfully detected in the liver of all diet groups, including six amino acids (l-glutamine, glycine, l-isoleucine, l-leucine, l-threonine, and l-serine), eight carbohydrates (α-D-galactopyranose, α-D-mannopyranoside, d-galactose, d-glucose, d-ribose, glucopyranose, d-mannose, and thymol-alpha-d-glucopyranoside), five organic compounds (malic acid, pentanedioic acid, butanedioic acid, silanamine, and 1,4-Butanediol) and two other metabolites (silanol and trisiloxane). Moreover, grouper fed with oleic acid diet produce the highest number of unique metabolites (10 metabolites). In this study, the unique metabolites refer to those metabolites identified only in a particular feeding group diet. Out of the unique metabolites extracted from the liver of grouper fed with oleic acid, the metabolites identified are from the fatty acid group comprising hexanedioic acid, 9,12-octadecadienoic acid, octadecanoic acid, oleic acid, and trans-13-octadecenoic acid. Other unique metabolites were identified from the amino acid group (aspartic acid, DL-ornithine, l-valine) and the inorganic compound group (carbonic acid and heptasiloxane).

As in the spleen samples (Fig. 5), 12 metabolites were successfully identified in all five diet groups, which include five from amino acid class (l-alanine, l-threonine, l-leucine, l-serine, and δ-aminolevulinic acid), three from carbohydrate class (d-galactose, d-glucose, and d-ribose) and four from other metabolite classes such as organic and inorganic metabolites (pentanedioic acid, silanamine, silanol, and 6-hydroxy-2-aminohexanoic acid). Contrary with the liver results, the highest number of unique metabolites were produced in grouper fed with a control diet (eight metabolites) followed by grouper fed with an oleic acid diet (five metabolites). The unique metabolites extracted from the grouper’s spleen fed with a control diet consisted of amino acids (cysteine, and l-methionine), carbohydrates (α-D-mannopyranoside, and d-fructose), fatty acid (propanoic acid), and organic compounds (pipecolic acid, pyrimidine, and phosphoric acid). Meanwhile, for grouper fed with oleic acid diet the metabolites identified are from the amino acid group comprising pyroglutamic acid, d-proline, and d-leucyl-d-leucine while from the organic compound group the metabolites comprising 4-aminomethylcyclohexane carboxylic acid and tris(trimethylsiloxy)ethylene.

### Metabolites distribution between the liver and spleen samples

**Figure 6:**
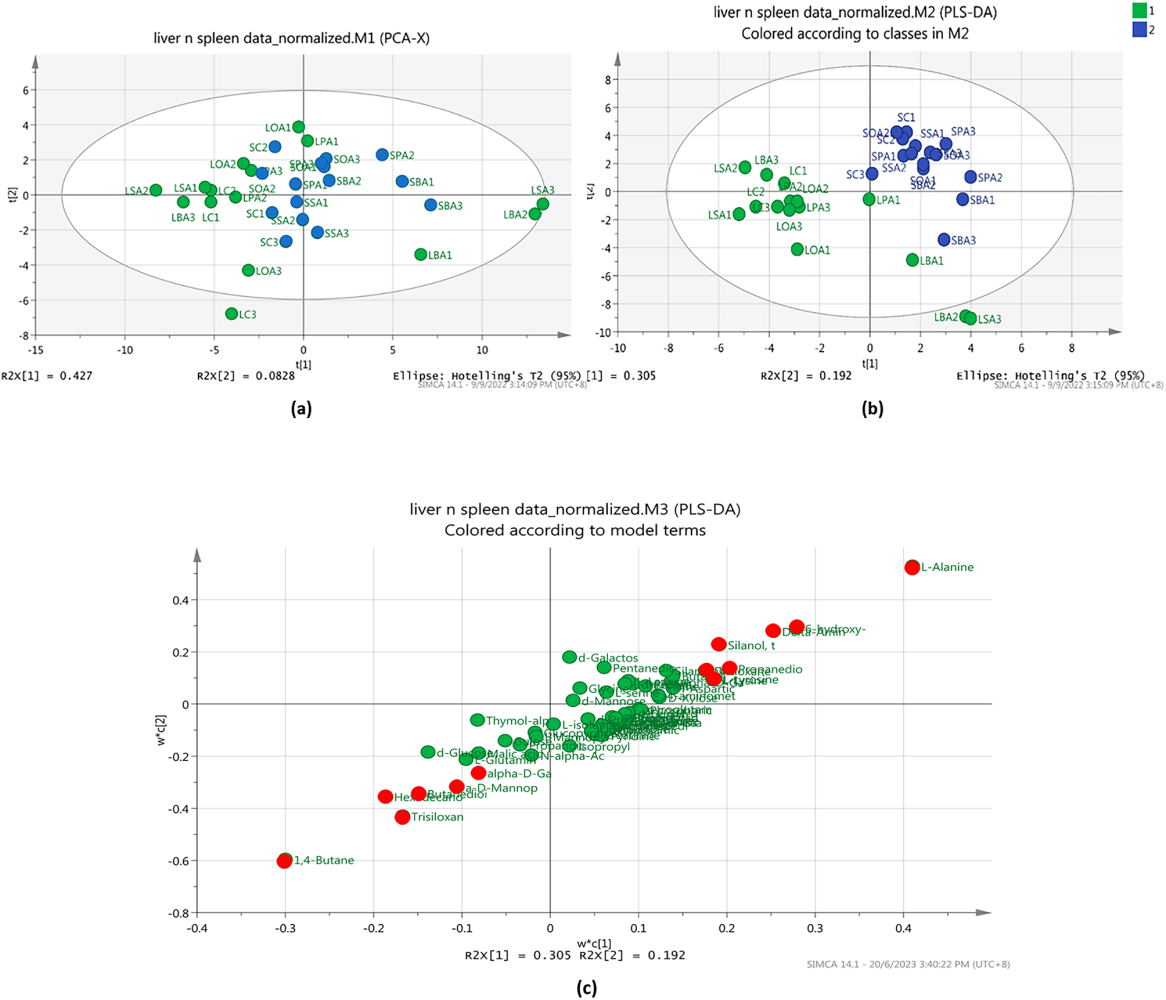
PCA score plot (**a**) and PLS-DA score plot (**b**) for liver and spleen metabolites depicting the separation pattern of two different organs. Liver sample (green), spleen sample (blue). PLS-DA loading plot analysis (**c**) of metabolites profiles from liver and spleen organ.

Comparisons between liver and spleen metabolites were performed with Principal component analysis (PCA) and partial least squares discriminant analysis (PLS-DA) score plot analysis. Here, a separation pattern between organs was observed via PCA and PLS-DA with the total variance percentage for PCA (R^2^X = 0.427, R^2^Y = 0.083) and PLS-DA analysis (R^2^X = 0.305, R^2^Y = 0.192) were 51.0% and 49.7% respectively. As refer to Fig. 6a, the PCA plot showed no distinct clusters between liver and spleen metabolites after challenged with *V. vulnificus*. Moreover, further analysis using PLS-DA plot as in Fig. 6b, shows discriminating between the liver and spleen metabolites. This observation suggested that metabolites are produced uniquely between the liver and spleen in response to *Vibrio* infection. Distant to model (DModX) analysis were carried out for the liver and spleen data, as LBA2 and LSA3 were observed to separated from others. From the result, the DModX value was observed to be lower than D-Crit with probability of 95% confidence interval indicates the samples were found to belong to the model sample group and not outliers. The variable influence on projection (VIP) was generated form a PLS-DA analysis. Figure 6c was used to identify metabolites that are significant for the differences in liver and spleen organs. Figure 6c shows the major metabolites with the VIP score value greater than one contribute to the separation between organs, including L-alanine, 1,4-butanediol, hexadecanoic acid, α-D-mannopyranoside, propanedioic acid, L-lysine, L-tyrosine, butanedioic acid, α-D-galactopyranose, 6-hydroxy-2-aminohexanoic acid, and silanol.

### Metabolites distribution among five different feeding groups in the liver and spleen samples

**Figure 7:**
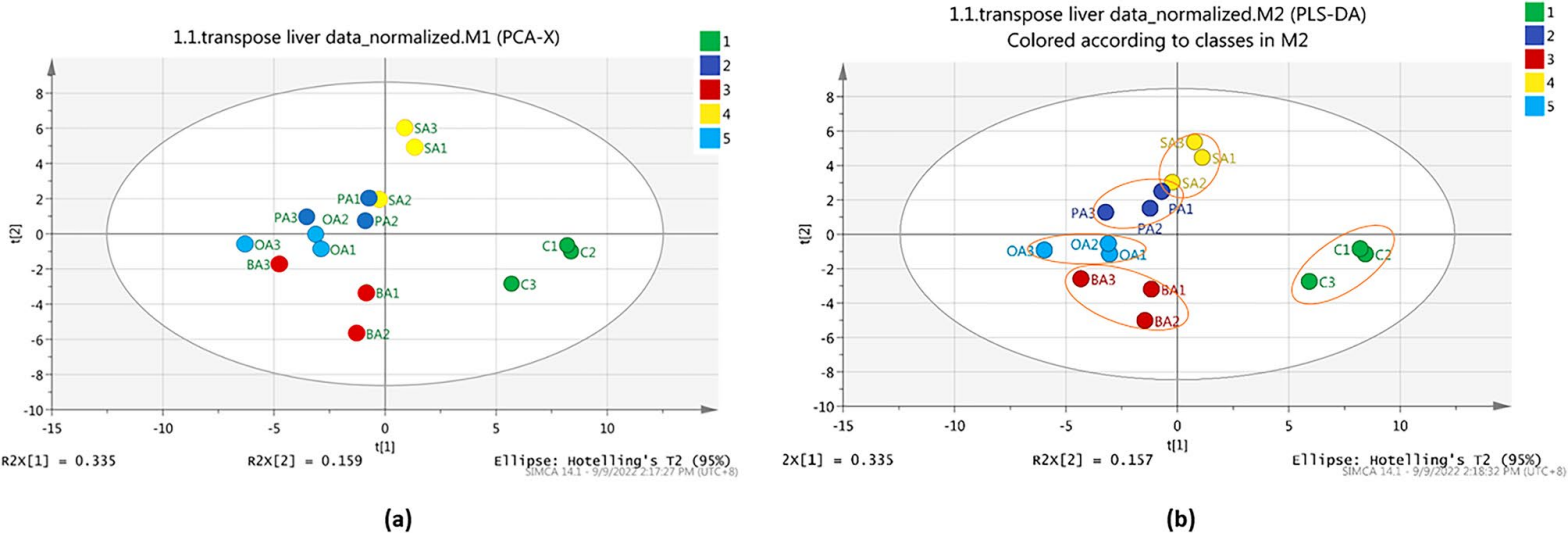
PCA score plot (**a**) and PLS-DA score plot (**b**) for liver metabolites depicting the separation pattern of five different sample groups. 1 (green)—control (infected and fed with control diet), 2 (dark blue)—PA (infected and fed with palmitic acid), 3 (red)—BA (infected and fed with behenic acid), 4 (yellow)—SA (infected and fed with stearic acid), and 5 (light blue)—OA (infected and fed with oleic acid).

**Figure 8:**
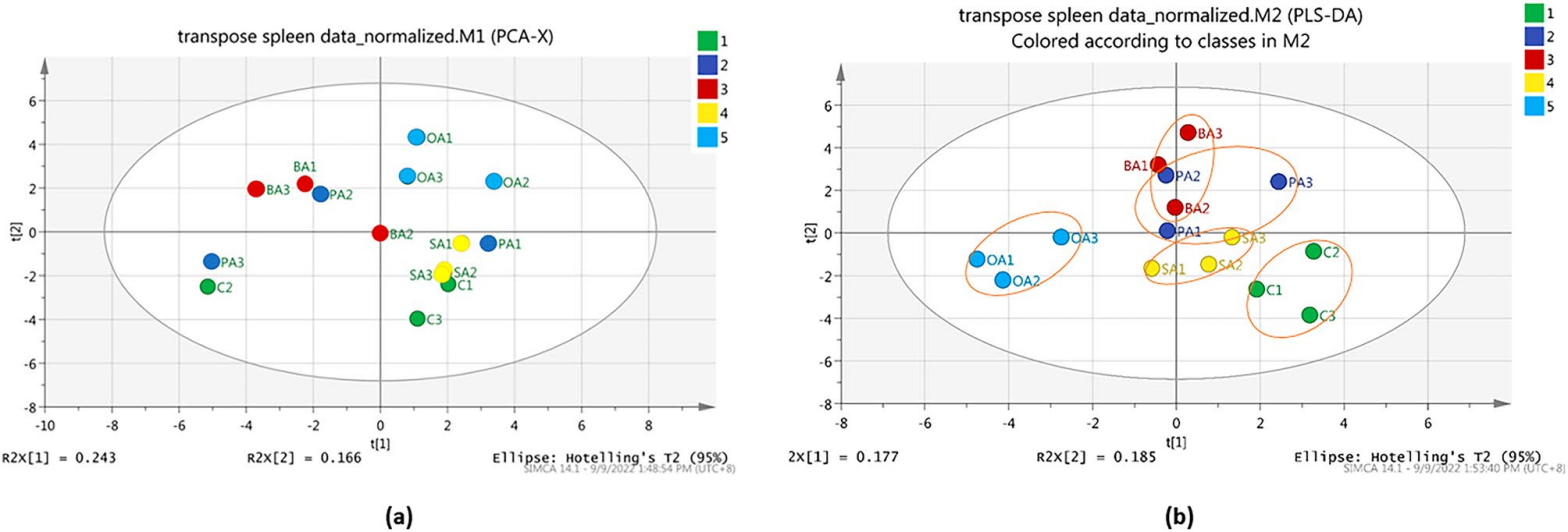
PCA score plot (**a**) and PLS-DA score plot (**b**) for spleen metabolites depicting the separation pattern of five different sample groups. 1 (green)—control (infected and fed with control diet), 2 (dark blue)—PA (infected and fed with palmitic acid), 3 (red)—BA (infected and fed with behenic acid), 4 (yellow)—SA (infected and fed with stearic acid), and 5 (light blue)—OA (infected and fed with oleic acid).

Further analyses using PCA and PLS-DA score plot for liver and spleen were carried out to determine the discrimination patterns among five different diet groups. As shown in Figs. 7a,b, a similar clear separation between feeding groups was observed via PCA and PLS-DA analysis with the total variance percentage of the liver for PCA (R^2^X = 0.335, R^2^Y = 0.159) and PLS-DA (R^2^X = 0.335, R^2^Y = 0.157) were 49.4% and 49.2% respectively. As for the spleen samples, Fig. 8a showed that the PCA score plot is not clustered properly as may be due to the small size of spleen samples which cause the quantity of extracted metabolites were reduced and identification of metabolites were more diversified. Here, the PCA score plot result had a total variance percentage (R2X) of 40.9% with the first PC (PC1) score value of 0.243, while the second PC (PC2) had a value of 0.166. However, a supervised method or a PLS-DA analysis was then further conducted, and the result showed a distinguish separation between five diet groups with a total variance percentage of 36.2% (RX2 = 0.177, R2Y = 0.185) (Fig. 8b).

By referring to the liver and spleen PLS-DA score plot results (Figs. 7b and 8b), three distinct clusters were observed among the survived-infected groupers fed with five different formulated diets. Specifically, in the liver samples, the palmitic acid diet group was clustered with the stearic acid diet group, the behenic acid diet group clustered with the oleic acid diet group, whereas the control diet group was separated farther away. Meanwhile, for the spleen sample, palmitic acid was clustered with behenic acid and stearic acid diet, whereas the oleic acid group and control group were separated farther away. It was observed that the metabolites produced by survived-infected groupers fed with an oleic acid diet are unique as they were distributed further from the other fatty acid diets, particularly if compared with the control diet. This observation suggested grouper fed with an oleic acid diet formulation contributed to the activation of the immune response during *Vibrio* infection. The finding was comparable to our previous results, where among different fatty acid diets tested on the fish, oleic acid formulation diet showed significant higher immune response activity through immunology assays, indicates oleic acid can elevated the fish immune response^22^.

**Figure 9:**
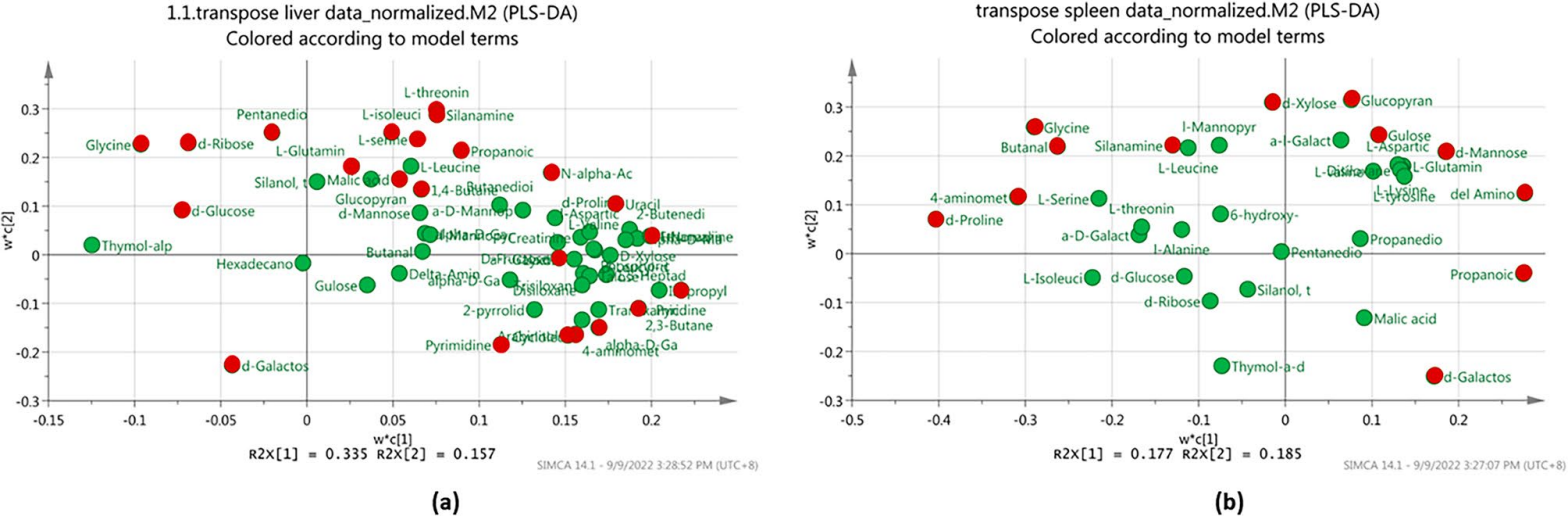
PLS-DA derived loading plot analysis of liver (**a**) and spleen (**b**) metabolites for five different diet groups. Compounds marked red indicate metabolites with a VIP value of more than 1.

The metabolites that influence the separation trend in the score plots can be found in Fig. 9. Discrimination metabolites were obtained from normalized data using a statistically significant threshold for Variable Importance in Projection (VIP) values obtained from the PLS-DA model. The p-values were calculated by two-way analysis of variance (ANOVA). The scores of more than one for liver and spleen metabolites are marked red as in Fig. 9.

Refer to Table 1, there are 23 compounds with a VIP score of more than one in the liver and 12 compounds with a VIP score of more than one in the spleen. The VIP score value more than one represents the variable’s contribution in estimating and distinguishing metabolites between the sample classes, which is in this study are the different types of formulated diet. Four classes of metabolites are present in both the liver and spleen organs, including glycine (amino acid), d-galactose (carbohydrate), propanoic acid (fatty acid), 4-aminomethylcyclohexane carboxylic acid, and silanamine (organic compound). The PLS-DA loading plot in Fig. 9a,b indicates the discernment of VIP metabolites that contributes to the differences among feeding treatment in the liver and spleen samples.

**Table 1:** Putatively identified metabolites in the liver and spleen samples with variable important for projection (VIP) score value exceeding 1 as determined by a PLS-DA analysis. Four biological replicates were pooled for metabolite extraction, and three technical replicates were used for GC–MS analysis.

No	Liver	VIP score	Spleen	VIP score
1	L-serine	1.571	D-proline	1.720
2	L-isoleucine	1.539	Glycine	1.557
3	Pyrimidine	1.468	Butanal	1.374
4	L-threonine	1.448	4-aminomethylcyclohexane carboxylic acid	1.365
5	D-glucose	1.444	Glucopyranose	1.317
6	Propanoic acid	1.411	δ-amino levulinic acid	1.303
7	D-ribose	1.403	D-xylose	1.237
8	Silanamine	1.352	D-galactose	1.194
9	1,4-butanediol	1.327	D-mannose	1.192
10	Glycine	1.270	Propanoic acid	1.166
11	D-fructose	1.250	Gulose	1.100
12	L-glutamine	1.187	Silanamine	1.012
13	α-D-mannopyranoside	1.121		
14	4-aminomethylcyclohexane carboxylic acid	1.112		
15	Pentanedioic acid	1.110		
16	2,3-butanediol	1.106		
17	D-Galactose	1.096		
18	Malic acid	1.054		
19	Arabinitol	1.032		
20	N-α-acetyl-L-lysine	1.031		
21	Pyridine	1.022		
22	α-D-galactofuranoside	1.007		
23	Uracil	1.000		

### Changes in metabolites by hierarchical clustering analysis

**Figure 10:**
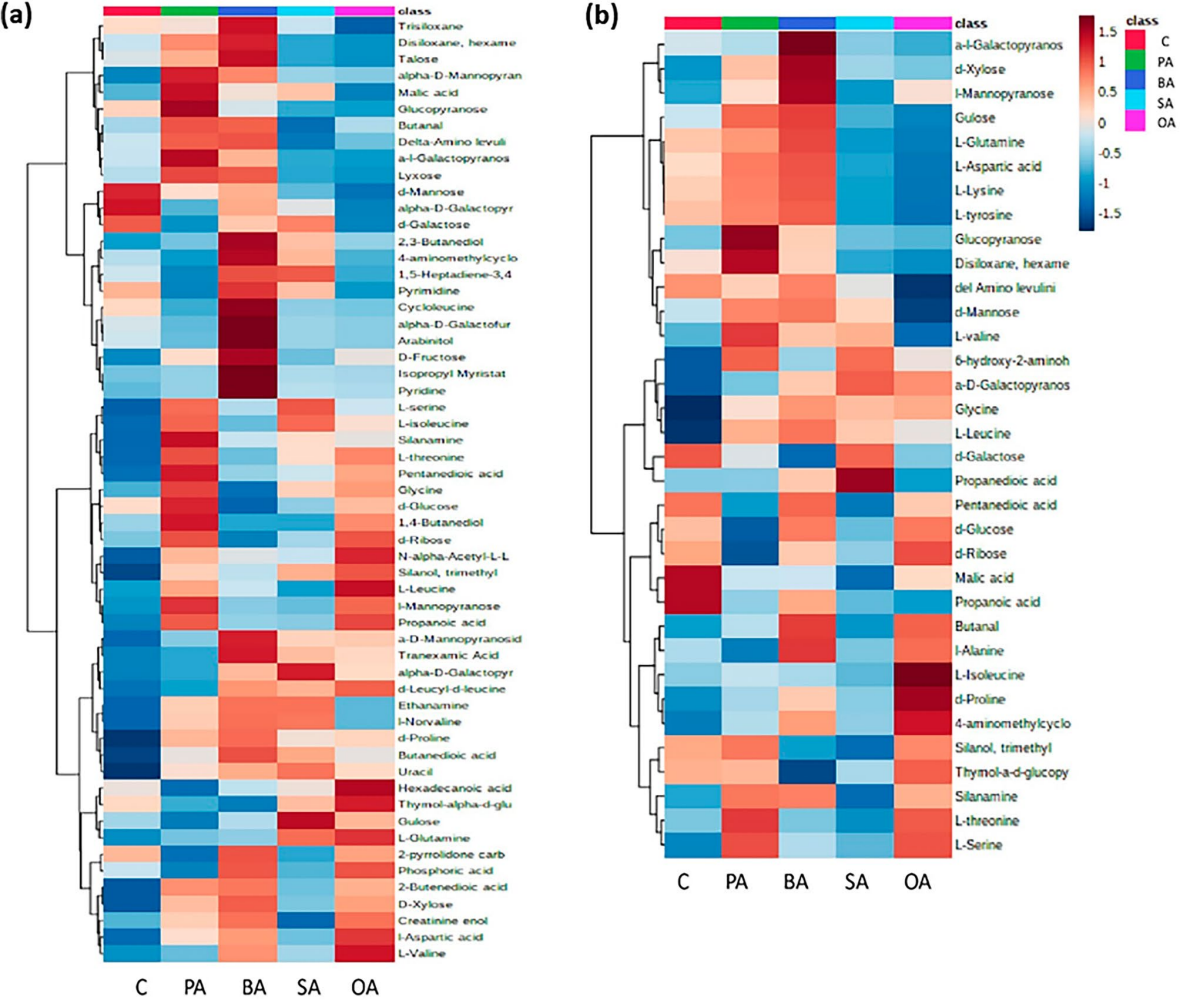
Hierarchical clustering analysis of detected metabolite compounds from the liver (**a**) and spleen (**b**) of survived-infected groupers fed with five different feed formulations. Brown color indicates relatively high abundance, blue represents a relatively low abundance.

Multivariate analysis was performed by hierarchical clustering analysis (heatmap) to examine the variation of metabolites expressed in the liver and spleen of all the survived-infected grouper fed with five different formulated diets (Fig. 10). The obvious difference can be found between the control group and other fatty acid groups. The grouper that fed with dietary fatty acids (oleic acid, palmitic acid, stearic acid and behenic acid) were observed to have relatively high intensities of metabolites compared to the control group in the liver samples (Fig. 10a). Similarly, in the spleen samples, the grouper fed with dietary fatty acids (oleic acid diet, palmitic acid and behenic acid) were observed to have relatively high intensities of metabolites compared to the control group diet, except for the grouper fed with the stearic acid diet which has the lowest intensity of metabolites than other feeding groups (Fig. 10b).

### Metabolic pathways enrichment of grouper’s immune response fed with five different diet group

**Figure 11:**
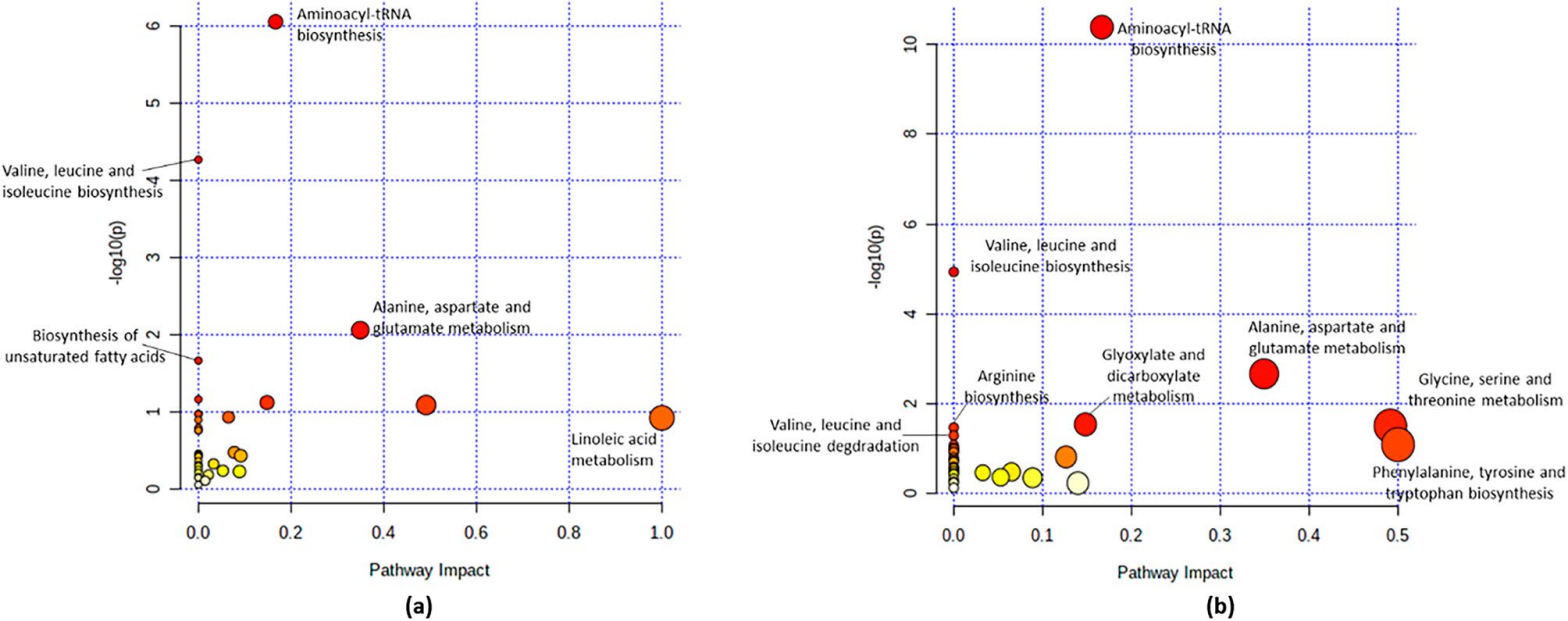
Pathway impact and statistical significance of metabolic pathways identified by pathway enrichment analysis of the metabolites. Liver sample (**a**); spleen sample (**b**). Y-axis shows a negative logarithm of the p-value (*p* < 0.05), indicates pathways with higher statistical significance are drawn higher in the graph.

The enrichment of metabolic pathways analysis was performed in the liver and the spleen of survived-infected grouper using MetaboAnalyst 5.0 and subjected to the KEGG pathway database. The statistical analysis of metabolic pathways was set at *p* < 0.05 for significant difference of differential metabolites and pathway impact values at 0.1 > 1.0. The impact value is the pathway impact value calculated from the pathway topology analysis. Hence, when compared the pathway impact value (0.1 > 1.0), linoleic acid metabolism was observed to have the highest impact value for liver (Fig. 11a), while in the spleen, phenylalanine, tyrosine, and tryptophan biosynthesis was observed to has the highest impact value (Fig. 11b).It was known that the metabolic pathway with impact an value > 0.1 are consider the most relevant pathways involved in the conditions under the study.

From thirty-three potential metabolic pathways that were identified in the liver samples (Supplementary Table 2), four pathways show enriched significant difference (*p* < 0.05) based on the differential metabolites, including aminoacyl-tRNA biosynthesis, valine, leucine and isoleucine biosynthesis, alanine, aspartate, and glutamate metabolism and biosynthesis of unsaturated fatty acids (Table 2). Meanwhile, in the spleen samples, from thirty-four potential metabolic pathways that were identified (Supplementary Table 3), seven pathways showed a significant difference (*p* < 0.05) enriched based on the differential metabolites, including aminoacyl-tRNA biosynthesis, valine, leucine, and isoleucine biosynthesis, alanine, aspartate, and glutamate metabolism, glyoxylate and dicarboxylate metabolism, glycine, serine, and threonine metabolism and arginine biosynthesis (Table 2).

**Table 2:** Metabolic pathways with significantly different responses based on the metabolites present in the liver and spleen samples of the survived-infected grouper. Only pathways with FDR (false discovery rate) < 0.1 are shown. Significant difference *p* < *0.05*.

	Metabolic pathway	p-value	–log 10 (p)	FDR	impact value
	Liver				
1	Aminoacyl-tRNA biosynthesis	8.8 × 10^−7^	6.06	7.4 × 10^−5^	0.17
2	Valine, leucine, and isoleucine biosynthesis	5.4 × 10^−5^	4.27	2.2 × 10^−1^	0.00
3	Alanine, aspartate, and glutamate metabolism	0.01	2.06	0.24	0.35
4	Biosynthesis of unsaturated fatty acids	0.02	1.67	0.45	0.00
5	Arginine biosynthesis	0.07	1.16	0.89	0.00
6	Glyoxylate and dicarboxylate metabolism	0.08	1.12	0.89	0.15
7	Glycine, serine, and threonine metabolism	0.08	1.09	0.89	0.49
8	beta-Alanine metabolism	0.11	0.97	0.89	0.00
9	Pantothenate and CoA biosynthesis	0.11	0.97	0.89	0.00
10	Pentose and glucuronate interconversions	0.12	0.93	0.89	0.06
11	Linoleic acid metabolism	0.12	0.92	0.89	1.00
12	Valine, leucine, and isoleucine degradation	0.13	0.90	0.89	0.00
13	Propanoate metabolism	0.16	0.79	0.97	0.00
14	D-Glutamine and D-glutamate metabolism	0.17	0.76	0.97	0.00
15	Nitrogen metabolism	0.17	0.76	0.97	0.00
	Spleen				
1	Aminoacyl-tRNA biosynthesis	4.1 × 10^−11^	10.39	3.4 × 10^−9^	0.167
2	Valine, leucine and isoleucine biosynthesis	1.2 × 10–5	4.93	0.001	0.00
3	Alanine, aspartate, and glutamate metabolism	2.0 × 10^−1^	2.66	0.06	0.35
4	Glyoxylate and dicarboxylate metabolism	0.03	1.53	0.49	0.15
5	Glycine, serine, and threonine metabolism	0.03	1.50	0.49	0.49
6	Arginine biosynthesis	0.04	1.46	0.49	0.00
7	Valine, leucine, and isoleucine degradation	0.05	1.28	0.63	0.00
8	Phenylalanine, tyrosine, and tryptophan biosynthesis	0.08	1.08	0.79	0.50
9	Propanoate metabolism	0.09	1.07	0.79	0.00
10	Lysine degradation	0.10	1.00	0.79	0.00
11	Glycolysis / Gluconeogenesis	0.11	0.97	0.79	0.00
12	D-Glutamine and D-glutamate metabolism	0.12	0.91	0.79	0.00
13	Nitrogen metabolism	0.12	0.91	0.79	0.00
14	Cysteine and methionine metabolism	0.16	0.80	0.90	0.13
15	Phenylalanine metabolism	0.16	0.79	0.90	0.00
16	Ubiquinone and other terpenoid-quinone biosynthesis	0.18	0.75	0.94	0.00
17	Biotin metabolism	0.20	0.71	0.95	0.00
18	Amino sugar and nucleotide sugar metabolism	0.20	0.69	0.95	0.00

However, notably, among these pathways, alanine, aspartate, and glutamate metabolism exhibited significant difference (*p* < 0.05) with the highest impact value of 0.35 in the liver (Table 2). Whereas, in the spleen, glycine, serine, and threonine metabolism exhibited significant difference (*p* < 0.05) with the highest impact value of 0.45 (Table 2). These results suggest that alanine, aspartate, and glutamate metabolism pathway in the liver and glycine, serine, and threonine metabolism pathway in the spleen play roles in fish resistance to *Vibrio* infection.

### Metabolic pathways enrichment of grouper’s immune response fed with five different diet groups

**Figure 12:**
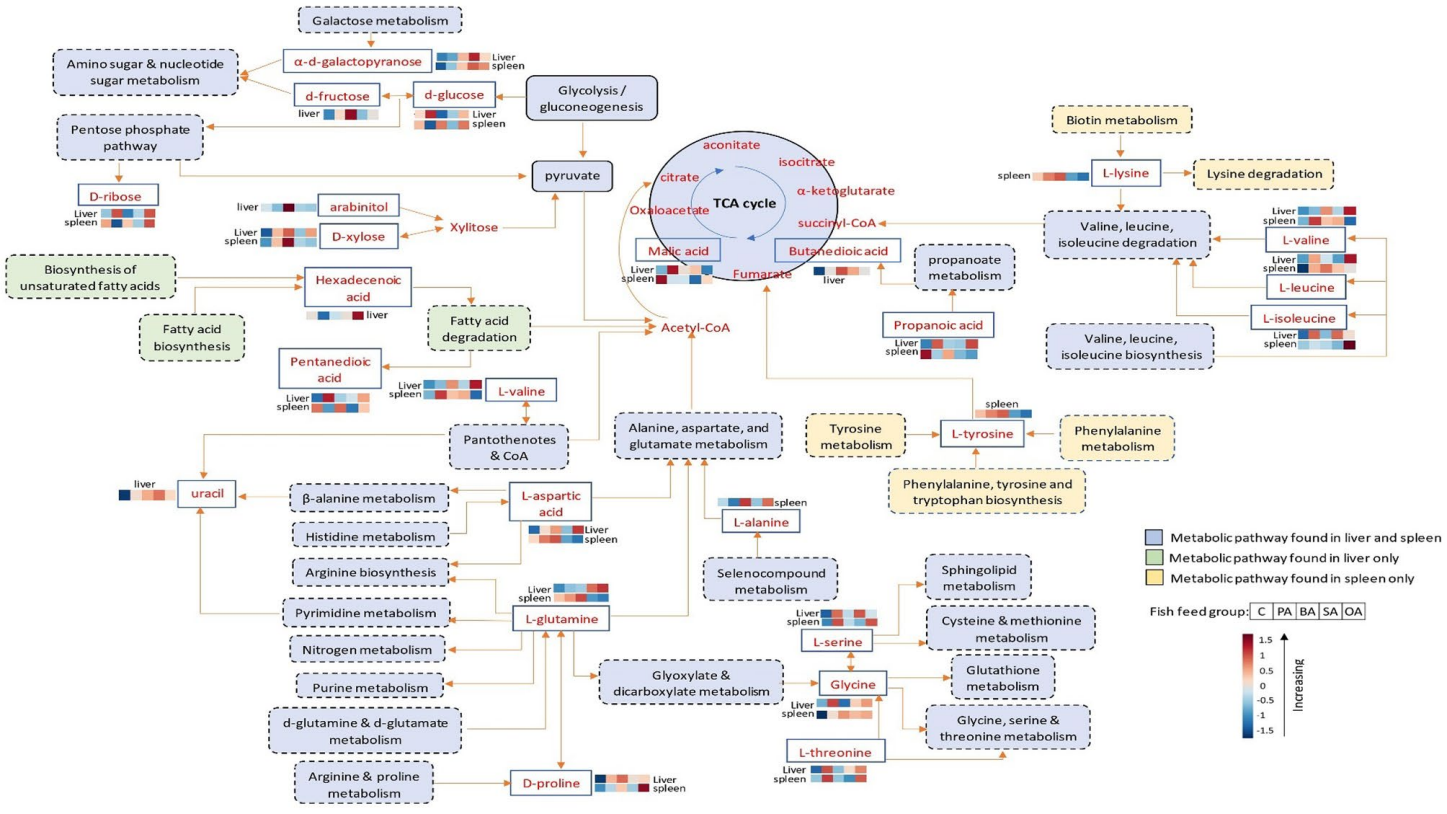
Integrated pathways contribute to the grouper’s immune response towards *Vibrio* infection based on the metabolite’s intensities in the liver and spleen samples of the survived-infected grouper. Blue represents pathways found in the liver and spleen that contributed to the grouper’s survival. Green represents pathways contributed to the grouper survival found in the liver only. Orange represents pathways contributed to the grouper survival found in the spleen only. Blue and red boxes represented increased and decreased intensities of metabolites in the liver and spleen samples for the survived-infected grouper fed with five different fish diets. One box represents the average replicates used in the study and their corresponding intensities.

Based on Fig. 12, the pathway map shows the relevant metabolic pathways networks together with the corresponding differential metabolites intensities in the liver and spleen samples from the survived-infected grouper fed with five different types of diet formulation. The high metabolites intensities can be observed in grouper fed with fatty acid diet formulation groups, while low metabolites intensities can be observed in grouper fed with control diet group. These data suggest that the control and fatty acid diets have differential metabolomes abundance possibly related to fish immune responses. Among these enriched pathways shown in Fig. 12, alanine, aspartate, and glutamate metabolism were identified as the significant and most impacted pathway (Table 2). This is in agreement with the high abundance of l-glutamine and aspartic acid metabolites found in the liver of the survived-infected grouper fed with the oleic acid diet. For spleen samples, enriched pathway namely glycine, serine, and threonine metabolism was shown to have the most impacted pathway between the significant pathways (Table 2). This result is in line with the high abundance of glycine, l-threonine, and l-serine in the spleen sample of groupers fed with oleic acid diet compared to the control diet as shown in Fig. 12.

## Discussion

In this study, four fatty acids namely oleic acid, palmitic acid, behenic acid, and stearic acid were used as immunostimulants in fish feed diet formulation. It has been known that fatty acids are necessary for fish to meet their specific nutrient requirement at different life stages^27^. In addition, fatty acids were able to manipulate immune responses by initiating various processes including gene regulation, membrane fluidity, lipid peroxidation, and eicosanoid production to make up the cell membrane^28^. In our study, the effects of fish feed formulation were observed and investigated to compare the relationship between metabolic changes and immunostimulant administration. Several studies also had showed that the used of immunostimulant could affects the fish metabolic activities and simultaneously elevating their immune responses toward infectious diseases^29–32^. The liver and spleen were chosen for the metabolomics analysis as the organs are adjacent linked to gut-associated lymphoid tissue (GALT) that produces varieties of immune and non-immune related enzymes. Moreover, their involvement in the hematopoiesis, production of antibodies, and antigen degradation process during immune response activities also interest us to use the liver and spleen in our study^33,34^.

Based on our previous results, grouper fed with dietary oleic acid, palmitic acid, stearic acid and behenic acid give higher survival rates compared to grouper fed with the control formulated diets toward *Vibrio* infection^22^. This result is consistent with a study of oleic acid as an anti-inflammatory agent that plays a role in activating various immune-competent cellular pathways^35^ and is responsible for modulating wound inflammation by inducing wound healing^36^. In our study, other ingredients such as fish meal and soybean meal have a function as protein sources, while vegetable oil is the lipid source. A study by Faudzi et al.^37^ found that soybean meal concentration in hybrid grouper’s daily feed uptake could reach 50% of the total feed formulation without significantly affecting hybrid grouper growth or body condition. The carbohydrates used in the study, such as corn flour, are a source of energy and can be used for binding activity in feed production. Vitamins and minerals are organic and inorganic compounds necessary for the normal growth and development of bodily functions in fish^27^. With the complete and optimal dietary components including proteins, carbohydrates, lipids, vitamins, and minerals in all diet group, the metabolomes changes in the grouper were assured not due to these components, but attributable to the addition of different fatty acids used which acts as an immunostimulant to elevate the fish immune response.

The supplementation of fatty acid compounds in general and clinically has been investigated in many different animal models and tissue cultures. In fish, several studies have mainly focused to elevate the health of fish, particularly on investigating the effect of the specific compound on diseases resistances and strengthening immunity^29,38,39^. For example, a study by Ebrahimi et al.^29^ focus on the use of organic acids for the growth of red hybrid tilapia, a study by Nayak et al.^38^ focus the use of docosahexaenoic and eicosapentaenoic acid (DHA/EPA) on the growth and immune response of golden pompano while, a study by Zhang et al.^39^ focus on the use of ω6 LC-PUFA-rich algae to improve zebrafish immune response toward *Streptococcal* infection. In another previous study, a high abundance of omega-9 fatty acids such as oleic acid, palmitoleic acid, 6,9-octadecenoic acid, 8,11-eicosadienoic acid, and cis-erucic acid had been identified to play a huge role in the grouper's immune response after *Vibrio* infection. Additionally, other fatty acids from the unsaturated fatty acid group including palmitic acid, stearic acid, arachidic acid, behenic acid, and lignoceric acid also show high metabolites intensities after *Vibrio* infection^21^. Hence, in our study, oleic acid, stearic acid, behenic acid, and palmitic acid were used as feed immunostimulants to stimulate the grouper immune response toward vibriosis infection. Control feed was prepared without the addition of any fatty acid. Four different fatty acids were chosen not only based on their positive finding in various aquatic species^20,22,40–42^ but also on their affordability as a fish feed ingredient that could reduce the fish feed production costs.

The metabolomic approach provides enormous potential insight in the aquaculture industry, not only for biomarker identification and disease diagnosis, but also contributes to understanding the biochemical pathways involving the fish immunological responses^23,43^. In our current study, GC–MS analysis was conducted on the liver and spleen samples of survived-infected grouper fed with five different formulated diets. After six weeks of feeding trial and one week of the post-bacterial challenge, the liver samples of the survived-infected grouper fed with oleic acid formulated diet showed higher metabolite compounds detection compared to the other fish formulated diets (Fig. 3). The higher metabolites identified in the oleic acid group might indicate that grouper required and regulated more metabolites to maintain their body from pathogen invasion^44^. In the current study, the metabolite changes in the survived-infected grouper from different feed formulation diet groups might be due to the pathogen's action to modify the metabolic activities of the grouper in resisting invasion^45^. In addition, the lesser metabolites detected in the spleen might be due to the minuscule size of the spleen compared to the liver, where it produced lesser metabolites as the spleen might be started to deteriorate and be incapable of fully functioning after *Vibrio* attacked the organism internal organs^46^.

From Fig. 3, metabolites from the amino acid group were produced greater in the liver of grouper fed with oleic acid formulated diet . However, in the spleen, amino acid metabolites were observed to be higher in the control diet group. Previous research stated that the amino acids play a major role in fish metabolism, especially during fish dietary protein conversion into fish protein, where high regulation of amino acids is required to substantial the role in energy metabolisms including gluconeogenesis (production of glucose from non-carbohydrate carbon substrates), lipogenesis (conversion of fatty acids and glycerol into fats), and oxidation substrates in fish^47^. As shown in Supplementary Table 2, the high abundance of amino acids was majorly found in the liver sample of survived-infected grouper fed with an oleic acid diet. These amino acids comprising of l-valine (1.8%), l-glutamine (1.8%), N-α-acetyl-l-lysine (1.7%), l-threonine (0.9%), and l-leucine (0.7%). Meanwhile, in the liver of survived-infected grouper fed with the control diet, glycine (13.7%) was identified as the highest abundance of amino acid compared to the oleic acid diet (7.9%). In comparison, the spleen samples have the highest abundance of amino acids comprising glycine (20.9%), l-alanine (3.2%), and l-leucine (0.6%) in the grouper fed with an oleic acid diet. Meanwhile. grouper fed with a control diet, l-glutamine (6.4%), l-aspartic acid (3.6%), and l-serine (1.4%) were found to be in high abundance in the spleen samples. These differences in percent area between diets might be attributed due to different reactions of each diet group to the metabolic mechanism of fish immune response to *Vibrio* infection after six weeks. The differences of percent area between feeding diet groups were not correlated to the number of metabolites produced in each diet group, but instead reflected the intensity or upregulate and downregulate of metabolites produced for each diet group^48^.

Looking at Fig. 5, most of the higher unique metabolites can be observed in liver samples from surviving infected grouper fed oleic acid, palmitic acid, behenic acid, and stearic acid diets. However, if we compare liver and spleen samples from surviving infected grouper fed with control diet, unique metabolites are much more abundant in the spleen (eight metabolites) than in the liver (one metabolite). This condition may be due to the need of grouper to release more metabolites to resist pathogen invasion while maintaining their survival, since no immunostimulant was added to their feed formulation in the control diet. The unique metabolites then act as signaling molecules that prompt the fish’s defense mechanisms.

Among these amino acids namely , l-leucine, -valine, and l-alanine, they have already proven to be significantly involved in the immune response of several fish species including the tilapia *Oreochromis niloticus* against *Streptococcus iniae*^49^, zebrafish *Danio rerio* against *V. alginolyticus*^50^ and Atlantic salmon against *Aeromonas salmonicida* infection^51^ . In tilapia, the present of exogenous l-leucine helps tilapia to modulate their metabolome to enhance innate immunity during *S. iniae* infection^49^. Meanwhile, for zebrafish effected with *V. alginolyticus*, valine and leucine were among different metabolites identified between survival and dying groups, suggested these metabolites involved in fish survival^50^. As for l-alanine, it was found to have function in inhibition of apoptosis and stimulating the proliferation of lymphocytes of Atlantic salmon during infection with *A. salmonicida* bacterial^51^. In another study, leucine, isoleucine, valine, and threonine showed highly metabolite abundance in the tissue muscle of the brown-marble grouper in response to *V. vulnificus* infection^45^. Research has revealed that l-valine promotes the macrophages’ phagocytosis to inhibit bacterial growth^52^. A previous study reported that amino acid such as glutamine is an important substrate in rapidly dividing cells including lymphocytes and enterocytes while producing energy for other intercellular transport, tissue growth, and cell migration^53^. Additionally, glutamine caused significant increases in erythrocytes’ function, which is known to have an essential role in transporting oxygen and carbon dioxide during fish respiration^54^. The efficiency of oxygen and carbon dioxide transport in the fish metabolism is also particularly important during the TCA cycle where glutamine will be converted to α-ketoglutarate and provide additional energy for the activation of fish immune responses^55^. Glycine has been found to stimulate the immune response of Nile tilapia challenged with *S. iniae* by elevating the myeloperoxidase activity which produces a number of reactive oxidant species (ROS) that is an important component in the innate immune response^56^. Meanwhile, it was revealed that the overproduction of ROS during the early infection in fish can be maintained with the presence of serine that helps in downregulating the ROS production which has a role in the activation of inflammasome and interleukin-1β in the macrophage^57^. According to Wu^44^, the requirements of amino acid as the key metabolic pathway in achieving the desired efficiency of metabolic transformation are important in the growth and maintaining the survival of the organisms during controlling infectious diseases. The high amino acid content in the current result agrees with the previous finding reported by Yang et al.^58^, where they successfully profiled a higher percentage of amino acids in the survived-infected zebrafish, *D. rerio* against *V. alginolyticus* infection compared to other metabolites groups.

Carbohydrate is a necessary component in the fish diet that provides energy to the whole-body tissues and organs. In fish, a constant supply of energy needs particularly if fish experience stress during pathogen infection. This energy is produced through the glycolysis process, in which glycogen is converted to glucose and use to initiate the immune mechanisms^59^. Glycolysis primarily occurs in the liver, where the accumulation of carbohydrates is in the form of glycogen^60^. In this study, the highest abundance of carbohydrate metabolites can be found in the liver samples of grouper fed with behenic acid formulated diet namely d-galactose (58.2%) and d-glucose (11.0%). Meanwhile, the lowest percent of d-galactose (41.5%) and d-glucose (7.5%) can be found in the liver samples of grouper fed with an oleic acid diet. (Supplementary Table 1). As in the spleen, carbohydrates such as d-glucose (10.2%), d-ribose (3.7%), thymol-α-d-glucopyranoside (8.3%), and α-d-galactopyranosiduronic acid (11.6%) were highly abundant in grouper fed with oleic acid formulated diet. Meanwhile, for grouper fed with stearic acid formulated diets, d-galactose (45.9%) and d-mannose (4.7%) were observed to have the highest percentage of carbohydrate metabolites compared to the other fish diet groups. The results were in line with the previous reports, which metabolites such as d-galactose, d-glucose, d-ribose^45^, and mannose^61^ were involved in elevating the fish immune mechanisms. The high glucose content in the liver and spleen samples indicates that fish have converted necessary components of carbohydrates through glycolysis from their diets into glucose to meet energy needs, thereby initiating fish immune mechanisms that enable fish to resist infectious diseases^60^. In addition, glucose has proven to play an important role in enhancing the immune response of tilapia fish against *E. tarda* infection^62^. According to Al-Banaw et al.^61^, mannose is produced richly in the mucus of catfish, *Arius tenuispinis* with other carbohydrates compositions including N-acetylgalactosamine and N-acetyl-glucosamine. It is known that the mucus containing antibacterial enzymes and innate immunity-like proteins on the fish skin surface serves as an important component in providing a physical and chemical barrier against pathogens invasion^63^. Moreover, d-galactose and mannose were identified to be in abundance in the skin mucus of gilthead seabream, *Sparus aurata* containing numerous immune components. In this context, galactose and mannose inhibited remarkably bacterial adhesion to the gill during *V. alginolyticus* infection^64^. In another study, it was mentioned that d-ribose forms a key part of many structures involved in the immune system. For example, ribose combination with guanine or thymine is converted to a ribofuranosyl which is known as an immune response-enhancing agent that improves the ability of Sir2p (silent information regulator protein) protein in given the rise for superior reactive oxygen species against pathogen infection^65–67^.

Among the feeding diet groups, groupers fed with the oleic acid diet were observed to have a high fatty acid abundance in their samples. Three metabolites from the fatty acid group comprised of oleic acid (1.5%), trans-13-octadecenoic acid (1.4%) and hexadecenoic acid (1.3%) were detected in high abundance in grouper’s liver fed with oleic acid diet (Supplementary Table 1). Although in the current study, not many metabolites from the fatty acid group were identified, the supplementation of fatty acids such as oleic acid, stearic acid, palmitic acid, and behenic acid in the fish feed formulation diet provided an important diet component to the fish, not only to maintain their structural cell membranes but also for extensive metabolic process and precursors to signalling molecules within the fish body^68,69^. Moreover, the survival rate of grouper fed with these fatty acid diets showed an increment compared to the control feed diet. Among these fatty acid diets, oleic acid was revealed to elevate the highest immune responses including lysozyme activity, respiratory burst activity and phagocytic activity of hybrid grouper infected with *V. vulnificus*^22^. According to Özogul & Özogul^70^, oleic acid is a monounsaturated fatty acid (MUFA) from the omega-9 fatty acids group, which could synthesize prostaglandin to help induce wound healing. In a previous study, oleic acid played a role as an anti-inflammatory component that activated different pathways of immune-competent cells^71^. Furthermore, in another study, the large yellow croaker fish, *Larimichthys polyactis* fed with olive oil formulated diet containing a high oleic acid composition of oleic acid shows an incremental pro-inflammatory gene expression and increasing activity for other immune-related proteins and enzymes, such as tumour necrosis factor-α (TNFα), interleukin-1β (IL-1β), and cyclooxygenase-2 (COX-2)^40^. Hence, the finding supported our results, where fatty acid supplementation such as oleic acid could help grouper maintain their survival and increase immunity by achieving desirable metabolic transformation during pathogen admission. According to Fadjar et al.^72^, oleic acid can react with the bacterial membranes, where oleic acid compound could break the lipoglycopeptides or glycodepsipeptides of a cell wall and kill the bacteria simultaneously, reducing the mortality rate. In the current study, an abundance of hexadecenoic acid in the liver of the grouper fed with oleic acid was in line with the result from previous report, which mentioned that hexadecenoic acid was found to acquire an anti-inflammatory activity that is comparable with other omega-3 fatty acids^73^.

The SIMCA-P + , PCA, and PLS-DA analyses were performed to analyse the distribution of metabolite compounds detected in the survived-infected grouper. As shown in Fig. 6b, a good cluster separation between liver and spleen samples indicated that metabolites are produced uniquely between the liver and spleen samples in response to *Vibrio* infection. When compared among the different feeding formulation, five distinct cluster discrimination pattern was observed between the five feeding groups namely oleic acid, stearic acid, behenic acid, palmitic acid and control in the liver, and spleen samples (Figs. 7b and 8b respectively). The results demonstrated that the liver and spleen samples from survived-infected groupers fed with dietary oleic acid had the largest effects based on their differential metabolites, as the oleic acid group was clustered farther from the control diet groups. Meanwhile, it is true that samples producing similar metabolites will naturally cluster, and samples producing different metabolites are more widely distributed, it is also important to note that concentration and sample size also plays an important role in determining the clustering pattern^74^.

Figure 12 shows the pathways enrichment of the liver and spleen samples from the survived-infected grouper. In our study, the metabolic profile, as well as metabolic pathways were carried out to explore the shared metabolites and pathways from five different formulated diets. In fact, previous studies reported that metabolites detection and pathways construction are involved in immune response mechanisms^75–77^. Among the significant (*p* < 0.05) pathways shown in Table 2, we identified two amino acid metabolism pathways with the highest impact values, including alanine, aspartate, and glutamate metabolism (impact = 0.35) for liver and glycine, serine, and threonine metabolism (impact = 0.49) for spleen organs, suggesting that these amino acid pathways play a role in fish survival during *V. vulnificus* infection.

Although we can observe in Table 2 that glycine, serine, and threonine metabolism pathway in the liver and phenylalanine, tyrosine and tryptophan biosynthesis in the spleen showed higher impact values of 0.49 and 0.50, respectively, however because there was no enriched significant difference (*p* > 0.05) between their differential metabolites, we determined that they did not have any effect on the fish immune response, as it is important that the p-value need to be at *p* < 0.05 and impact value at 0.1 > 1.0 . Our results are consistent with the results reported by Baharum et al.^78^ and Yang et al.^79^. In Baharum et al.^78^ study, it has been revealed that alanine, aspartate, and glutamate metabolism is one of the crucial pathways that have a protective role in increasing ATP production considering that l-glutamine can enter the TCA cycle and provide energy to drive the defence mechanisms against infectious diseases^50^. Meanwhile, Yang et al.^79^ have reported that glycine, serine, and threonine metabolism was identified to be the most impacted pathway that elevated the immune response of Nile tilapia, *O. niloticus*, after being infected with *E. tarda*. Here, the serine metabolite identified from the head kidney sample of Nile tilapia was reported to have the highest metabolites abundance among the other identified metabolites. This is consistent with a previous study^80^, where serine promotes the production of Interleukin 1β (IL-1β) in macrophages and acts as a key mediator for the inflammatory response, which is essential in the activation of the fish immune response towards pathogen invasion. In addition, serine has been shown to have the ability to maintain homeostasis by balancing ROS production, where persistently high levels of ROS can lead to hyperactivation of immune responses leading to tissue damage^79,81^.

In line with our previous finding, where an oleic acid diet effectively boosts the grouper immune response^22^, our current results reported that l-glutamine and l-aspartic acid metabolites which belong to the most impacted pathway of alanine, aspartate, and glutamate metabolism were highly abundant in the liver samples of grouper fed with the oleic acid diet compared to the control diet group (Fig. 12). Meanwhile, l-serine, glycine, and l-threonine metabolites which belong to the most impacted pathway of glycine, serine, and threonine metabolism were highly abundant in the spleen samples of grouper fed with oleic acid compared to the control diet group (Fig. 12). This finding has proved that the roles of metabolites such as l-glutamine, l-aspartic acid, l-serine, glycine, and l-threonine in regulating the metabolic pathways can increase the immune response of groupers fed with an oleic acid diet against *V. vulnificus* infection. A similar study revealed a high abundance of amino acid content including threonine, glutamine, and aspartic acid were found in the survived-infected grouper with *V. vulnificus*^45,78^.

## Conclusion

In this work, GC–MS based analysis approach was used to characterize the metabolome changes in the liver and the spleen samples of survived-infected grouper fed with five different types of diet formulation. The metabolome changes in the survived-infected grouper were due to the response of grouper towards *Vibrio* infection depending on the fatty acid diet consumption and not because of the malnutrition mentioned in the discussion. Based on the PLS-DA-derived analysis, the metabolites distinction clustering between grouper fed with the oleic acid diet and grouper fed with the control diet suggests the oleic acid diet provides an impact on the activation of the grouper immune response after *Vibrio* infection. The oleic acid diet increased the alanine, aspartate, and glutamate metabolism pathway as well as the abundance of l-glutamine (1.8%) and l-aspartic acid (0.6%) metabolites. These amino acids were detected highly abundance in the liver of grouper fed with the oleic acid diet compared to the control diet. Meanwhile, in the spleen, glycine, serine, and threonine metabolism pathway were increased in line with the high abundance of glycine (20.9%), l-threonine (1.0%), and l-serine (0.8%) in grouper fed with the oleic acid diet compared to the control diet. Therefore, we propose this metabolic modulation as a promising approach to modify the grouper's immune response mechanisms, which give a great potential to activate the immune response and increase the survival of the grouper.

## Methods

### Fish experimental design

**Figure 13:**
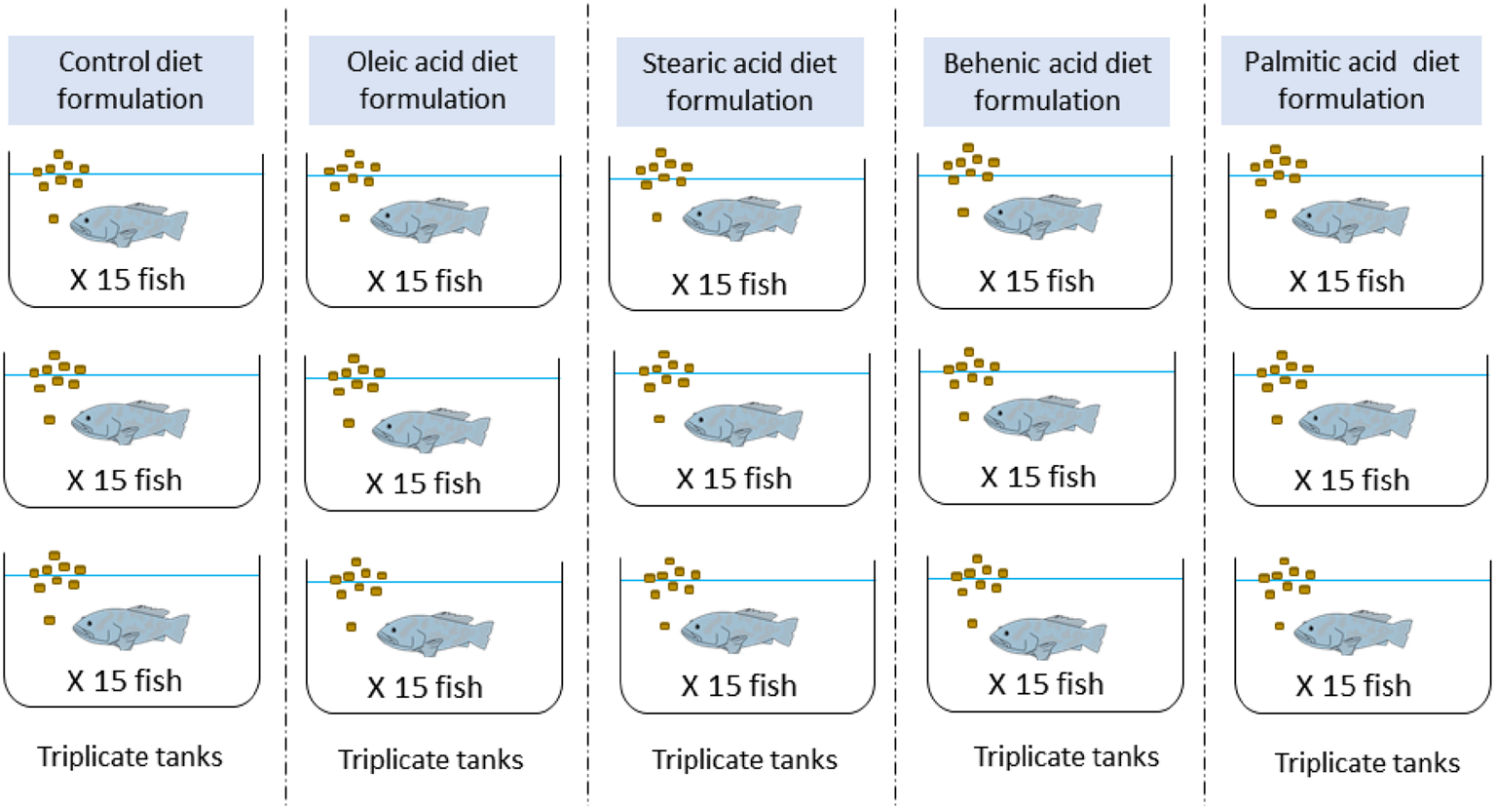
Diagram of aquaria tank set-up for five different diet formulations.

The experiment was carried out in the experimental facilities at Hatchery Unit, Institute of Bioscience, Universiti Putra Malaysia. All experiments were conducted following guidelines and regulations approved by the Universiti Kebangsaan Malaysia Animal Ethical Committee (UKMAEC) (Reference number: IBC/Ack/2/2019). The reporting in the manuscript follows the recommendations in the ARRIVE guidelines. Two hundred and fifty groupers with a length and weight of approximately 3.0–4.0 inches and 11.5 ± 0.5 g respectively were obtained from the local hatchery farm of Pantai Dasar Sabak, Kota Bahru, Kelantan. The grouper was acclimatized for one week in a 1000 L fiberglass tank filled with filtered seawater and equipped with water filter pump system and aeration. The water quality was maintained during the acclimatization period at temperature 28.0 ± 1 °C, pH around 8.0 ± 1, salinity around 30 ± 1 ppt and dissolved oxygen (DO) at ranged between 5.5 and 6.0 mg/L using YSI Multiprobe System. After initial acclimation period, the 225 groupers were then assigned to 15 glass aquaria equally (3n = 15 fish/ 60 × 60 × 40 cm glass aquaria), where each was filled with approximately 80 L of filtered seawater. Each diet group was represented in three replicates (Fig. 13).

Further acclimation was carried out for five days before specific feeding supplementation was given to the grouper. Five different diet formulations were prepared based on Natnan et al.^22^ feed formulation (Table 3). The grouper’s feed formulation includes the supplementation of oleic acid, stearic acid, behenic acid, and palmitic acid in each of the formulated diet preparation, while the control diet was without the addition of any fatty acid supplementation. The grouper was fed at 9.00 am and 4.00 pm daily for six weeks. During the feeding experiment, each glass aquaria was supplied with aeration while water quality parameters were maintained at 28.0 ± 1 °C, salinity at 30 ± 1 (ppt), pH at 8.0 ± 1, and DO at ranged between 5.5 and 6.0 mg/L using YSI Multiprobe System. The water exchange was conducted every two days exchanging approximately 50% of the total volume.

**Table 3:** Fish feed ingredient formulation (%). Oleic acid (R&M Chemicals 112,801); stearic acid (R&M Chemicals 57,114); behenic acid (Alfa Aesar A12850); palmitic acid (R&M Chemicals 57,103); α-Cellulose (Sigma-Aldrich Co. C8002). Proximate analysis was determined according to the 20th Edition of Association of Official Analytical Chemists (AOAC) by UniPEQ, Sdn. Bhd.

Ingredients	Control	Oleic acid	Stearic acid	Behenic acid	Palmitic acid
Fish meal	11.5	11.5	11.5	11.5	11.5
Soybean meal	50.5	50.5	50.5	50.5	50.5
Vegetable oil	7.5	7.5	7.5	7.5	7.5
Corn flour	24.0	24.0	24.0	24.0	24.0
Vitamin mixture	2.0	2.0	2.0	2.0	2.0
Mineral mixture	2.0	2.0	2.0	2.0	2.0
Fatty acids	0.0	2.0	2.0	2.0	2.0
α-Cellulose	2.5	0.5	0.5	0.5	0.5
Total	100	100	100	100	100
Proximate composition					
Protein	31.3	31.4	31.0	30.8	31.3
Carbohydrate	45.7	43.7	44.1	42.7	43.2
Lipid	9.4	11.4	11.3	11.1	11.2
Total ash	6.9	6.7	6.8	6.7	6.7
Moisture	6.7	6.8	6.8	8.7	7.6
Total	100	100	100	100	100
Energy (kcl/100 g)	393	403	402	394	399

### Fish challenged

*V. vulnificus* was revived from the glycerol stock obtained from infected grouper^21^. Identification of bacterial strains was conducted as previous study by Natnan et al.^22^. A single colony of *V. vulnificus* was picked from a thiosulphate citrate bile salt sucrose (TCBS) plate and cultured overnight in tryptic soy broth (TSB) at 30° for 18–24 h. The bacterial culture was then adjusted to a concentration of 5.4 × 10^7^ CFU/mL with fresh TSB medium using spectrometry. After six weeks of the feeding experiment, all groupers fed with different fatty acid diets including in the control group was infected with a median lethal dose (LD_50_) of *V. vulnificus* (5.4 × 10^7^ CFU/mL)^22^. After 30 min of immersion in *V. vulnificus,* the grouper fingerlings were transferred back into their perfective glass aquaria contained with clean seawater^21^. During this 30 min of constant exposure, bacteria can attach on fish skin and gills, while some were able to enter the fish organs. The feeding regime was resumed for another week. After one-week of post-bacterial challenge, the skin of the dead fish was swabbed, and the samples were streaked on the TCBS agar plates for *Vibrio* existence. The plates were incubated in 30 °C for 18 – 24 h. After one-week of post-bacterial challenge, the survival grouper from all five different feed diets were chosen for later GC–MS analysis.

### Sampling

After one week of the post-bacterial challenge, the survived-infected groupers were sacrificed before the liver samples were harvested and weighted individually. The average weighted for pooled liver samples were between 0.2 and 0.45 g and spleen were between 0.02 and 0.04 g. The samples were then pooled from four survived grouper fingerlings in each glass aquaria to represent one biological replicate. To ensure the minimum requirement in volume for metabolite extraction, the liver samples from the same glass aquaria were pooled together. The same harvesting process was done for the spleen samples. A total of four biological individuals were prepared for each five different feeding groups with three technical replicates. All samples were collected in the morning.

### Metabolite extraction

The pooled samples were quickly flash-frozen with liquid nitrogen and stored at − 80 °C prior metabolite extraction. The pooled organs were ground with liquid nitrogen separately for each replicate. The powdered sample was then subjected to metabolite extraction adapted from Mayalvanan^82^, and Wu et al.^83^ with modification. The sample that was ground with liquid nitrogen was mixed with the final ratio of 2.0:2.0:1.8 solvents (methanol: chloroform: water). During metabolite extraction, the solvents and samples are kept cold to avoid any degradation. The grounded sample was added with 4 mL/g of cold methanol and 1.6 mL/g of cold water mentioned earlier before vortexed. Then, 2 mL/g of cold chloroform was added to the mixture and vortexed. The mixture was incubated in ice for 40 min before centrifuging at 4 °C and 10 000 rpm for 10 min. After centrifugation, the supernatant was added with 2 mL/g of cold chloroform and 2 mL/g of cold water before the mixture was centrifuged again at 4 °C and 10 000 rpm for 20 min. The upper biphasic layer was then transferred into the new vial and stored at − 80 °C prior to GC–MS analysis. The steps were repeated for all harvested organs.

### Sample derivatization using TMS

The TMS derivatization method was based on the Roessner et al.^84^, and Azizan et al.^85^ method with modification. The addition of 2 µl internal standard (0.2 µmol of 10 mmol solutions of 2,3,3,3- d4 D, L-alanine) to each 100 µl sample of metabolite extraction was done. The supernatant was dried in a vacuum concentrator before the sample was resuspended in methoxyamine hydrochloride (MeOX) solution in pyridine (50 µL, 2 g/100 mL). The mixture was then incubated at 40 °C for 90 min. N-methyl-N-(trimethylsilyl)-trifluoroacetamide (MSTFA) was added (approximately 50 µL), followed by incubation at 40 °C for 30 min. The final incubation sample was then transferred to a new GC–MS vial for GC–MS analysis.

### GC–MS sample analysis

The GC–MS analysis was performed based on the method by Nurdalila et al.^45^, and Azizan et al.^85^ with some modifications. The GC–MS Turbo Mass Clarus 600 from Perkin Elmer coupled to an electron ionisation (EI) operated at 70 eV quadruple with a mass selective detector was used for the sample analysis. An aliquot of about 1.0 μL sample was injected into an Elite-5 MS capillary column (0.25 μm thickness × 30.0 m × 0.25 mm i.d.) that was coated with dimethylpolysiloxane (95%) and crosslinked diphenyl (5%) in the split mode of 50:1. The temperature for injection was set at 250 °C, while the ion source temperature was set to 200 °C. A continuous helium gas flow with the rate of 1.1 mL per min was supplied at GC temperatures ranging between 70 °C and 300 °C. The full scan mode of *m/z* 45–600 was used as the acquired spectra.

### Mass spectrometry data processing and data analysis

The raw data from the network common data form (Net-CDF) format were converted for GC–MS analysis. GC–MS data table with retention time, metabolite name, peak area, match, and the relative match was generated using TurboMass™ GC–MS software (Perkin Elmer, USA). The identification of metabolites was carried out using the NIST (National Institute of Standards and Technology) mass spectral database library (NIST 2008) with a match cut-off of 800 values discarded from the data. The internal standard and all other metabolites corresponding to the solvents were excluded prior to multivariate analysis. The value of the peak area was used to represent the detected metabolites. Data was than normalized against the internal standard followed by sum log transformed and Pareto scaling. Two-way Analysis of Variance (ANOVA) using MetaboAnalyst 5.0 server was used to statistically validate the values (https://www.metaboanalyst.ca/MetaboAnalyst/ModuleView.xhtml) with significance levels of *p* < 0.05 between independent groups. The normalized and validated data was then exported to SIMCA-P + version 12.0 (Umetrics AB, Umea, Sweden) for visualization and validated data using the Principal Component Analysis (PCA) and Partial Least Squares Discriminant Analysis (PLS-DA) model. The heatmap with hierarchical clustering analysis was also performed.

### Metabolic biosynthetic pathway mapping

Pathway enrichment analysis was performed using MetPa and the Kyoto Encyclopedia of Genes and Genomes (KEGG) to visualize the interactions of metabolic pathways. Lists of metabolites present in the spleen and liver of survived-infected grouper fed with control and fatty acid diets were uploaded, and pathways corresponding to the metabolite lists were extracted for both sets of samples. These pathways were then matched with those listed in KEGG to visualize the pathways interaction with each other. A comparative analysis was performed to identify unique pathways in the survived-infected grouper fed with the oleic acid diet. Relevant signaling pathways were integrated and appropriately mapped to understand the metabolic adaptations of survived-infected grouper fed with different fatty acid diets to *Vibrio* infection.

### Supplementary Information

Supplementary Information 1.

Supplementary Information 2.

Supplementary Information 3.

## Data Availability

All data generated or analyzed during this study are included in this published article (and its Supplementary Information files).

## References

[1] FAO. Statistic, Food and Agriculture Organization of the United Nations. *Rome, Italy* http://www.fao.org/fishery/statistics/en (2020).

[2] Cheng SS, Senoo S, Siddiquee S, Rodrigues KF (2015). Genetic variation in the mitochondrial genome of the giant grouper *Epinephelus lanceolatus* (Bloch, 1790) and its application for the identification of broodstock. Aquac. Rep..

[3] Mustafa, S. Climate change adaptation in aquaculture in Sabah: Strategic choices and imperatives. *CCIR News* 12 (2012).

[4] Bunlipatanon P, U-taynapun K (2017). Growth performance and disease resistance against *Vibrio vulnificus* infection of novel hybrid grouper ( *Epinephelus lanceolatus* × *Epinephelus fuscoguttatus* ). Aquac. Res..

[5] Ebi I, Lal TM, Ransangan J, Yong ASK, Shapawi R (2018). Susceptibility of hybrid grouper (*Epinephelus fuscogutattus* ♀ × *Epinephelus lanceolatus* ♂) to *Vibrio harveyi* VHJR7. AACL Bioflux.

[6] Zhu ZM, Dong CF, Weng SP, He JG (2018). The high prevalence of pathogenic Vibrio harveyi with multiple antibiotic resistance in scale drop and muscle necrosis disease of the hybrid grouper, *Epinephelus fuscoguttatus* (♀) × *E. lanceolatus* (♂), in China. J. Fish Dis..

[7] Mohamad N (2019). Natural concurrent infection of *Vibrio harveyi* and *V.* alginolyticus in cultured hybrid groupers in Malaysia. J. Aquat. Anim. Health.

[8] Shen GM (2017). Isolation, identification and pathogenicity of Vibrio harveyi, the causal agent of skin ulcer disease in juvenile hybrid groupers Epinephelus fuscoguttatus × Epinephelus lanceolatus. J. Fish Dis..

[9] Xu X (2017). Identification of pathogenicity, investigation of virulent gene distribution and development of a virulent strain-specific detection PCR method for *Vibrio harveyi* isolated from Hainan Province and Guangdong Province, China. Aquaculture.

[10] Amalina NZ (2019). Prevalence, antimicrobial susceptibility and plasmid profiling of Vibrio spp. isolated from cultured groupers in Peninsular Malaysia. BMC Microbiol..

[11] Hoihuan A (2021). Molecular genotyping and phenotyping of *Vibrio vulnificus* isolated from diseased, brown-marbled grouper (*Epinephelus fuscoguttatus*) in Thailand with preliminary vaccine efficacy analysis. Aquaculture.

[12] Abdullah, A. *et al.* Concurrent infections in tiger grouper (*Epinephelus fuscoguttatus*) cultured in deep sea cages in Langkawi. in *International Fisheries Symposium (IFS 2015)* (2015).

[13] Ina-Salwany MY (2019). Vibriosis in Fish: A review on disease development and prevention. J. Aquat. Anim. Health.

[14] Li J (2019). Effects of potential probiotic *Bacillus velezensis* K2 on growth, immunity and resistance to *Vibrio harveyi* infection of hybrid grouper (*Epinephelus lanceolatus *♂ × *E. fuscoguttatus *♀). Fish Shellfish Immunol..

[15] Heng S-P (2017). *Vibrio vulnificus*: An environmental and clinical Burden. Front. Microbiol..

[16] Vincent AT, Gauthier J, Derome N, Charette SJ, Derome N (2019). The rise and fall of antibiotics in aquaculture. Microbial communities in aquaculture ecosystems: Improving productivity and sustainability.

[17] Zhang Q (2021). Effects of dietary florfenicol contained feeds on growth and immunity of European seabass (*Dicentrarchus labrax*) in flow-through and recirculating aquaculture system. Aquac. Rep..

[18] Chowdhury S, Saikia SK (2020). Oxidative stress in Fish: A Review. J. Sci. Res..

[19] Mehana E, Rahmani A, Aly S (2015). Immunostimulants and fish culture: An overview. Annu. Res. Rev. Biol..

[20] Sankian Z, Khosravi S, Kim Y-O, Lee S-M (2019). Total replacement of dietary fish oil with alternative lipid sources in a practical diet for mandarin fish, *Siniperca scherzeri*, juveniles. Fish. Aquat. Sci..

[21] Nurdalila AA, Mayalvanan Y, Baharum SN (2019). Metabolite profiling of *Epinephelus fuscoguttatus* infected with vibriosis reveals Omega 9 as potential metabolite biomarker. Fish Physiol. Biochem..

[22] Natnan ME (2022). Comparison of different dietary fatty acids supplement on the immune response of hybrid grouper (*Epinephelus fuscoguttatus* × Epinephelus lanceolatus) challenged with *Vibrio vulnificus*. Biology (Basel).

[23] Natnan ME, Low C-F, Chong C-M, Bunawan H, Baharum SN (2021). Integration of omics tools for understanding the fish immune response due to microbial challenge. Front. Mar. Sci..

[24] Lulijwa R, Alfaro AC, Young T (2022). Metabolomics in salmonid aquaculture research: Applications and future perspectives. Rev. Aquac..

[25] Natnan ME (2021). Omics strategies in current advancements of infectious fish disease management. Biology (Basel).

[26] Low C-F, Rozaini MZH, Musa N, Syarul Nataqain B (2017). Current knowledge of metabolomic approach in infectious fish disease studies. J. Fish Dis..

[27] Craig, S., Helfrich, L. A., Kuhn, D. & Schwarz, M. H. Understanding fish nutrition, feeds, and feeding.e. *Virginia Coop. Ext.* 420–256 (2017).

[28] Al-Khalaifah H (2020). Modulatory effect of dietary polyunsaturated fatty acids on immunity, represented by phagocytic activity. Front. Vet. Sci..

[29] Ebrahimi M (2017). Comparing the effects of different dietary organic acids on the growth, intestinal short-chain fatty acids, and liver histopathology of red hybrid tilapia (*Oreochromis* sp.) and potential use of these as preservatives. Fish Physiol. Biochem..

[30] Wei C (2021). Dietary arachidonic acid supplementation improves the growth performance and alleviates plant protein-based diet-induced inflammation in juvenile turbot (*Scophthalmus maximus* L.). Aquac. Nutr..

[31] Ahmed I, Ahmad I (2021). Dietary lysine modulates growth performance, haemato-biochemical indices, non-specific immune response, intestinal enzymatic activities and antioxidant properties of rainbow trout, Oncorhynchus mykiss fingerlings. Aquac. Nutr..

[32] Dawood MAO, Koshio S, Esteban MÁ (2018). Beneficial roles of feed additives as immunostimulants in aquaculture: A review. Rev. Aquac..

[33] Trivedi PJ, Adams DH (2016). Gut–liver immunity. J. Hepatol..

[34] Lewis SM, Williams A, Eisenbarth SC (2019). Structure and function of the immune system in the spleen. Sci. Immunol..

[35] Jebali A (2020). Attenuation of inflammatory response in the EAE model by PEGlated nanoliposome of pistachio oils. J. Neuroimmunol..

[36] Ishak WMW, Katas H, Yuen NP, Abdullah MA, Zulfakar MH (2019). Topical application of omega-3-, omega-6-, and omega-9-rich oil emulsions for cutaneous wound healing in rats. Drug Deliv. Transl. Res..

[37] Mohd Faudzi N (2018). Soy protein concentrate as an alternative in replacement of fish meal in the feeds of hybrid grouper, brown-marbled grouper (*Epinephelus fuscoguttatus*) × giant grouper (*E. lanceolatus*) juvenile. Aquac. Res..

[38] Nayak S, Khozin-Goldberg I, Cohen G, Zilberg D (2018). Dietary supplementation with ω6 LC-PUFA-rich algae modulates zebrafish immune function and improves resistance to *Streptococcal* infection. Front. Immunol..

[39] Zhang M (2019). Effects of different dietary ratios of docosahexaenoic to eicosapentaenoic acid (DHA/EPA) on the growth, non-specific immune indices, tissue fatty acid compositions and expression of genes related to LC-PUFA biosynthesis in juvenile golden pompano Trachin. Aquaculture.

[40] Li X (2019). High level of dietary olive oil decreased growth, increased liver lipid deposition and induced inflammation by activating the p38 MAPK and JNK pathways in large yellow croaker (*Larimichthys crocea*). Fish Shellfish Immunol..

[41] Librán-Pérez M, Pereiro P, Figueras A, Novoa B (2019). Antiviral activity of palmitic acid via autophagic flux inhibition in zebrafish (*Danio rerio*). Fish Shellfish Immunol..

[42] Jubie S (2012). Synthesis, antidepressant and antimicrobial activities of some novel stearic acid analogues. Eur. J. Med. Chem..

[43] Amoah K (2022). Ultra-Performance Liquid Chromatography-Mass Spectrometry-Based untargeted metabolomics reveals the key potential biomarkers for castor meal-induced enteritis in juvenile hybrid grouper (*Epinephelus fuscoguttatus ♀* × *E. lanceolatus ♂*). Front. Nutr..

[44] Wu G (2014). Dietary requirements of synthesizable amino acids by animals: A paradigm shift in protein nutrition. J. Anim. Sci. Biotechnol..

[45] Nurdalila AA, Natnan ME, Baharum SN (2020). The effects of amino acids and fatty acids on the disease resistance of *Epinephelus fuscoguttatus* in response to *Vibrio vulnificus* infection. 3 Biotech.

[46] Amaro C (2015). The fish pathogen *Vibrio vulnificus* biotype 2: Epidemiology, phylogeny, and virulence factors involved in warm-water Vibriosis. Microbiol. Spectr..

[47] Li X, Zheng S, Guoyao W, Guoyao W (2021). Nutrition and functions of amino acids in fish. Amino Acids in Nutrition and Health: Amino Acids in the Nutrition of Companion, Zoo and Farm Animals.

[48] Aggio RB, Mayor A, Reade S, Probert CS, Ruggiero K (2014). Identifying and quantifying metabolites by scoring peaks of GC-MS data. BMC Bioinform..

[49] Du C (2017). Metabolic mechanism for L-leucine-induced metabolome to eliminate Streptococcus iniae. J. Proteome Res..

[50] Gong Q (2020). Metabolic modulation of redox state confounds fish survival against *Vibrio alginolyticus* infection. Microb. Biotechnol..

[51] Liu PF, Du Y, Meng L, Li X, Liu Y (2016). Metabolic profiling in kidneys of Atlantic salmon infected with *Aeromonas salmonicida* based on 1 H NMR. Fish Shellfish Immunol..

[52] Chen X (2017). Exogenous L-valine promotes phagocytosis to kill multidrug-resistant bacterial pathogens. Front. Immunol..

[53] Wu G (2009). Amino acids: Metabolism, functions, and nutrition. Amino Acids.

[54] Li H-T (2017). Dietary glutamine improves the function of erythrocytes through its metabolites in juvenile carp (*Cyprinus carpio var*. Jian). Aquaculture.

[55] Azeredo R, Serra CR, Oliva-Teles A, Costas B (2017). Amino acids as modulators of the European seabass, *Dicentrarchus labrax*, innate immune response: An in vitro approach. Sci. Rep..

[56] Xie S, Zhou W, Tian L, Niu J, Liu Y (2016). Effect of N-acetyl cysteine and glycine supplementation on growth performance, glutathione synthesis, anti-oxidative and immune ability of Nile tilapia *Oreochromis niloticus*. Fish Shellfish Immunol..

[57] Wang P (2019). Macrophage achieves self-protection against oxidative stress-induced ageing through the Mst-Nrf2 axis. Nat. Commun..

[58] Yang M-J (2018). Boosted TCA cycle enhances survival of zebrafish to *Vibrio alginolyticus* infection. Virulence.

[59] Soto-Heredero G, de las Gómez Heras MM, Gabandé-Rodríguez E, Oller J, Mittelbrunn M (2020). Glycolysis – a key player in the inflammatory response. FEBS J..

[60] Alarcon F, Martinez T, Diaz M, Moyano FJ (2001). Characterization of digestive carbohyrase activity in the gilthead seabream (*Sparus aurata*). Hydrobiologia.

[61] Al-Banaw A, Kenngott R, Al-Hassan JM, Mehana N, Sinowatz F (2010). Histochemical analysis of glycoconjugates in the skin of a catfish (*Arius tenuispinis*, Day ). Anat. Histol. Embryol..

[62] Zeng Z (2017). Glucose enhances tilapia against *Edwardsiella tarda* infection through metabolome reprogramming. Fish Shellfish Immunol..

[63] Dash S, Das SK, Samal J, Thatoi H (2018). Epidermal mucus, a major determinat in fish heatlh: A review. Iran. J. Vet. Res..

[64] Cerezuela R, Guardiola FA, Cuesta A, Esteban MÁ (2016). Enrichment of gilthead seabream (*Sparus aurata* L.) diet with palm fruit extracts and probiotics: Effects on skin mucosal immunity. Fish Shellfish Immunol..

[65] Lewin GR, Stacy A, Michie KL, Lamont RJ, Whiteley M (2019). Large-scale identification of pathogen essential genes during coinfection with sympatric and allopatric microbes. Proc. Natl. Acad. Sci..

[66] Seifert JG, Brumet A, St Cyr JA (2017). The influence of D-ribose ingestion and fitness level on performance and recovery. J. Int. Soc. Sports Nutr..

[67] Kenyon, K. E. Using D-ribose with or without anti-microbial agents to enhance healing and subsequent recovery by both synthesizing and sparing NAD derivatives. 10 (2003).

[68] Calder PC, Waitzberg DL, Klek S, Martindale RG (2020). Lipids in parenteral nutrition: Biological aspects. J. Parenter. Enter. Nutr..

[69] Taşbozan Oğuz, Gökçe Mahmut Ali, Catala Angel (2017). Fatty acids in fish. Fatty acids.

[70] Özogul Y, Özogul F (2007). Fatty acid profiles of commercially important fish species from the mediterranean, Aegean and black seas. Food Chem..

[71] Carrillo C, Cavia MM, Alonso-Torre S (2012). Role of oleic acid in immune system; mechanism of action: A review. Nutr. Hosp..

[72] Fadjar M, Andajani S, Zaelani K (2016). Squid (*Loligo edulis*) ink raw extract as an anti– vibriosis substance in grouper (*Epinephelus fuscoguttatus*) juvenile culture infected by *Vibrio alginolyticus*. AACL Bioflux.

[73] Guijas C, Meana C, Astudillo AM, Balboa MA, Balsinde J (2016). Foamy monocytes are enriched in cis -7-hexadecenoic fatty acid (16:1n–9), a possible biomarker for early detection of cardiovascular disease. Cell Chem. Biol..

[74] Allen F, Greiner R, Wishart D (2015). Competitive fragmentation modeling of ESI-MS/MS spectra for putative metabolite identification. Metabolomics.

[75] Zhang J, Pavlova NN, Thompson CB (2017). Cancer cell metabolism: The essential role of the nonessential amino acid, glutamine. EMBO J..

[76] Holeček M (2018). Branched-chain amino acids in health and disease: Metabolism, alterations in blood plasma, and as supplements. Nutr. Metab. (Lond).

[77] Li J (2018). Lysine 39 of IKKε of black carp is crucial for its regulation on IRF7-mediated antiviral signaling. Fish Shellfish Immunol..

[78] Baharum SN (2022). LC–qTOF-MS analysis of fish immune organs reveals the distribution of amino acids in response to metabolic adaptation of the survival phenotype in grouper against *Vibrio* infection. 3 Biotech.

[79] Yang DX (2021). Serine metabolism tunes immune responses to promote *Oreochromis niloticus* survival upon *Edwardsiella tarda* Infection. mSystems.

[80] Chen S (2020). Serine supports IL-1β production in macrophages through mTOR signaling. Front. Immunol..

[81] Lightfoot YL, Blanco LP, Kaplan MJ (2017). Metabolic abnormalities and oxidative stress in lupus. Curr. Opin. Rheumatol..

[82] Mayalvanan Y (2019). Metabolomics study of the response of Epinephelus fuscoguttatus towards vibriosis.

[83] Wu H, Southam AD, Hines A, Viant MR (2008). High-throughput tissue extraction protocol for NMR- and MS-based metabolomics. Anal. Biochem..

[84] Roessner U, Wagner C, Kopka J, Trethewey RN, Willmitzer L (2000). Simultaneous analysis of metabolites in potato tuber by gas chromatography-mass spectrometry. Plant J..

[85] Azizan KA, Baharum SN, Mohd Noor N (2012). Metabolic profiling of *Lactococcus lactis* under different culture conditions. Molecules.

