# Supplementary material for: Oleic acid as potential immunostimulant in metabolism pathways of hybrid grouper fingerlings (Epinephelus fuscoguttatus × Epinephelus lanceolatus) infected with Vibrio vulnificus
Source: Sci Rep. 2023 Aug 8;13:12830. doi: 10.1038/s41598-023-40096-7 (PMC10409752; doi:10.1038/s41598-023-40096-7)
Supplement: Supplementary file 1 — Supplementary Information 1. [file 41598_2023_40096_MOESM1_ESM.docx]

Supplementary Table 3 Pathway analysis based on the metabolites present in the spleen samples from the survived-infected grouper.

|  | **Metabolic pathway** | **p value** | **–log 10 (p)** | **FDR** | **impact value** |
| --- | --- | --- | --- | --- | --- |
| 1 | Aminoacyl-tRNA biosynthesis* | 4.05×10^-11^ | 1.04×10^1^ | 3.41×10^-9^ | 0.17 |
| 2 | Valine, leucine, and isoleucine biosynthesis* | 1.17×10^-5^ | 4.93 | 0.001 | 0.00 |
| 3 | Alanine, aspartate, and glutamate metabolism* | 2.00×10^-3^ | 2.66 | 0.06 | 0.35 |
| 4 | Glyoxylate and dicarboxylate metabolism* | 0.03 | 1.53 | 0.49 | 0.15 |
| 5 | Glycine, serine, and threonine metabolism* | 0.03 | 1.50 | 0.49 | 0.49 |
| 6 | Arginine biosynthesis* | 0.04 | 1.46 | 0.49 | 0.00 |
| 7 | Valine, leucine, and isoleucine degradation* | 0.05 | 1.28 | 0.63 | 0.00 |
| 8 | Phenylalanine, tyrosine, and tryptophan biosynthesis | 0.08 | 1.08 | 0.79 | 0.50 |
| 9 | Propanoate metabolism | 0.09 | 1.07 | 0.79 | 0.00 |
| 10 | Lysine degradation | 0.10 | 1.00 | 0.79 | 0.00 |
| 11 | Glycolysis / Gluconeogenesis | 0.11 | 0.97 | 0.79 | 0.00 |
| 12 | D-Glutamine and D-glutamate metabolism | 0.12 | 0.91 | 0.79 | 0.00 |
| 13 | Nitrogen metabolism | 0.12 | 0.91 | 0.79 | 0.00 |
| 14 | Cysteine and methionine metabolism | 0.16 | 0.80 | 0.90 | 0.13 |
| 15 | Phenylalanine metabolism | 0.16 | 0.80 | 0.90 | 0.00 |
| 16 | Ubiquinone and other terpenoid-quinone biosynthesis | 0.18 | 0.75 | 0.94 | 0.00 |
| 17 | Biotin metabolism | 0.20 | 0.71 | 0.96 | 0.00 |
| 18 | Amino sugar and nucleotide sugar metabolism | 0.21 | 0.69 | 0.96 | 0.00 |
| 19 | Nicotinate and nicotinamide metabolism | 0.26 | 0.58 | 1.00 | 0.00 |
| 20 | Histidine metabolism | 0.28 | 0.55 | 1.00 | 0.00 |
| 21 | Butanoate metabolism | 0.28 | 0.55 | 1.00 | 0.00 |
| 22 | beta-Alanine metabolism | 0.33 | 0.49 | 1.00 | 0.00 |
| 23 | Selenocompound metabolism | 0.33 | 0.49 | 1.00 | 0.00 |
| 24 | Pantothenate and CoA biosynthesis | 0.33 | 0.49 | 1.00 | 0.00 |
| 25 | Pentose and glucuronate interconversions | 0.34 | 0.47 | 1.00 | 0.07 |
| 26 | Citrate cycle (TCA cycle) | 0.36 | 0.45 | 1.00 | 0.03 |
| 27 | Sphingolipid metabolism | 0.37 | 0.43 | 1.00 | 0.00 |
| 28 | Pentose phosphate pathway | 0.38 | 0.42 | 1.00 | 0.00 |
| 29 | Galactose metabolism | 0.45 | 0.35 | 1.00 | 0.05 |
| 30 | Glutathione metabolism | 0.46 | 0.34 | 1.00 | 0.09 |
| 31 | Arginine and proline metabolism | 0.57 | 0.25 | 1.00 | 0.00 |
| 32 | Pyrimidine metabolism | 0.60 | 0.23 | 1.00 | 0.00 |
| 33 | Tyrosine metabolism | 0.61 | 0.22 | 1.00 | 0.14 |
| 34 | Purine metabolism | 0.77 | 0.11 | 1.00 | 0.00 |

*Significant difference p< 0.05
