# Supplementary material for: Oleic acid as potential immunostimulant in metabolism pathways of hybrid grouper fingerlings (Epinephelus fuscoguttatus × Epinephelus lanceolatus) infected with Vibrio vulnificus
Source: Sci Rep. 2023 Aug 8;13:12830. doi: 10.1038/s41598-023-40096-7 (PMC10409752; doi:10.1038/s41598-023-40096-7)
Supplement: Supplementary file 2 — Supplementary Information 2. [file 41598_2023_40096_MOESM2_ESM.docx]

**Supplementary Table 1.** List of identified metabolites from liver and spleen of survived-infected groupers fed with five different formulated diets. C represents control (feed without any key metabolites), OA represents grouper fed with oleic acid diet, PA represent grouper fed with palmitic acid diet, BA represents grouper fed with behenic acid diet, and SA represents grouper fed with stearic acid diet.

|  | Metabolites |  | Feeding groups | | | | | | | | | |
| --- | --- | --- | --- | --- | --- | --- | --- | --- | --- | --- | --- | --- |
|  |  | Groups | Liver | | | | | Spleen | | | | |
|  |  |  | C | OA | PA | BA | SA | C | OA | PA | BA | SA |
| 1 | 1,4-Butanediol | Organic compound | 0.12±0.03 | 0.14±0.03 | 0.18±0.07 | 0.08±0.03 | 0.10±0.03 | - | - | - | - | - |
| 2 | 1,5-Heptadiene-3,4-diol, 2-methyl- | Organic compound | 0.04±0.01 | - | - | - | 0.03±0.01 | - | - | - | - | - |
| 3 | 2-Butenedioic acid | Organic compound | - | 0.03±0.01 | 0.04±0.02 | - | - | - | - | - | - | - |
| 4 | 2,3-Butanediol | Organic compound | - | - | - | 0.04±0.01 | 0.03±0.01 | - | - | - | - | - |
| 5 | 2,4-Pentadienenitrile | Organic compound | - | - | - | 0.02±0.01 | - | - | - | - | - | - |
| 6 | 4-aminomethylcyclohexane carboxylic acid | Organic compound | 0.05±0.02 | - | - | 0.03±0.01 | 0.04±0.01 | - | 0.31±0.18 |  | - | - |
| 7 | 6-hydroxy-2-aminohexanoic acid | Carboxylic acid | - | - | 0.60±0.04 | - | - | 0.55±0.25 | 0.92±0.04 | 0.99±0.01 | 0.80±0.06 | 0.76±0.14 |
| 8 | 9,12-Octadecadienoic acid | Fatty acid | - | 0.40±0.02 | - | - | - | - | - | - | - | - |
| 9 | α-D-galactofuranoside | Carbohydrate | 0.05±0.02 | - | - | 0.05±0.02 | - | - | - | - | - | - |
| 10 | α-D-galactopyranosiduronic acid | Carbohydrate | - | 0.49±0.01 | - | - | 2.08±0.67 | - | 11.63±0.51 | - | - | 2.80±0.19 |
| 11 | α-D-galactopyranose | Carbohydrate | 0.75±0.02 | 0.26±0.01 | 0.56±0.04 | 0.13±0.04 | 0.48±0.01 | - | - | - | - | - |
| 12 | α -D-galactopyranoside | Carbohydrate | - | - | 0.19±0.01 | - | - | - | - | - | - | - |
| 13 | α-D-mannopyranose | Carbohydrate | - | - | 0.31±0.01 | - | - | - | - | - | - | - |
| 14 | α-D-mannopyranoside | Carbohydrate | 0.37±0.01 | 0.44±0.01 | 0.38±0.01 | 0.29±0.01 | 0.24±0.08 | 0.93±0.26 | - |  | - | - |
| 15 | α-l-galactopyranoside | Carbohydrate | 0.20±0.06 | - | 0.22±0.09 | - | - | 0.24±0.11 | - | - | 0.37±0.13 | - |
| 16 | α-l-mannopyranoside | Carbohydrate | - | - | 0.14±0.01 | - | - | - | - | - | - | - |
| 17 | δ-amino levulinic acid | Amino acid | 0.47±0.15 | 0.08±0.05 | 0.56±0.07 | 0.31±0.01 | - | 0.68±0.24 | 0.71±0.37 | 0.50±0.09 | 0.54±0.21 | 0.47±0.12 |
| 18 | Aminomalonic acid | Organic compound | - | - | - | 0.05±0.02 | - | - | - | - | - | - |
| 19 | Aspartic acid | Amino acid | - | 0.35±0.01 | - | - | - | - | - | - | - | - |
| 20 | Arabinitol | Sugar alcohol | 0.68±0.02 | - | - | 1.34±0.04 | - | - | - | - | - | - |
| 21 | Butanal | Aldehyde | 0.10±0.03 | 0.25±0.08 | 0.42±0.05 | 0.36±0.01 | - | 0.45±0.20 | 1.00±0.19 | 0.74±0.24 | 1.41±0.66 | - |
| 22 | Butanedioic acid | Organic compound | 0.11±0.03 | 0.11±0.06 | 0.12±0.09 | 0.20±0.07 | 0.10±0.03 | - | - | - | 0.25±0.09 | - |
| 23 | Butane | Alkane | - | - | - | - | 0.02±0.01 | - | - | - | - | - |
| 24 | Butanedinitrile | Nitrile | 0.04±0.03 | - | - | - | 0.03±0.02 | - | - | - | - | - |
| 25 | Carbonic acid | Inorganic compounds | - | 0.06±0.04 | - | - | - | - | - | - | - | - |
| 26 | Creatinine enol | Organic compound | 0.30±0.01 | 0.55±0.01 | 0.43±0.01 | - | - | - | - | - | - | - |
| 27 | Cysteine | Amino acid | - | - | - | - | - | 0.08±0.02 | - | - | - | - |
| 28 | Cycloleucine | Amino acid | 0.41±0.01 | - | - | 0.13±0.04 | - | - | - | - | - | - |
| 29 | d-fructose | Carbohydrate | - | 0.24±0.01 | 0.45±0.03 | 0.58±0.02 | - | 0.69± | - | - | - | - |
| 30 | d-galactose | Carbohydrate | 46.28±9.96 | 41.51±2.26 | 47.06±5.52 | 58.16±9.82 | 49.66±0.52 | 43.58±3.24 | 21.01±1.43 | 22.54±1.13 | 44.97±1.54 | 45.88±1.89 |
| 31 | d-glucose | Carbohydrate | 8.43±2.22 | 7.53±1.86 | 8.70±3.97 | 11.02±3.75 | 8.95±0.03 | 7.06±0.64 | 10.52±2.51 | 6.99±2.25 | 6.47±0.36 | 7.62±0.13 |
| 32 | d-mannose | Carbohydrate | 7.85±1.21 | 3.16±1.85 | 3.30±0.31 | 1.82±0.62 | 3.33±1.27 | 1.08±0.01 | - | 2.63±0.37 | 1.75±0,38 | 4.70±0.43 |
| 33 | d-proline | Amino acid | - | 0.40±0.01 | 0.76±0.02 | 0.09±0.03 | 0.15±0.06 | - | 0.37±0.04 | - | - | - |
| 34 | d-ribose | Carbohydrate | 1.33±0.02 | 1.92±0.75 | 1.95±0.81 | 1.51±0.51 | 1.64±0.12 | 2.86±0.16 | 3.65±0.99 | 2.34±0.78 | 3.35±0.16 | 2.19±0.14 |
| 35 | D-xylose | Carbohydrate | - | 0.17±0.05 | 0.17±0.07 | - | - |  | - | 0.49±0.18 | 1.92±0.20 | - |
| 36 | d-Leucyl-d-leucine | Amino acid | - | 0.18±0.06 | - | - | 0.04±0.02 | - | 0.15±0.04 | - | - | - |
| 37 | DL-ornithine | Amino acid | - | 0.11±0.07 | - | - | - | - | - | - | - | - |
| 38 | Dodecamethylcyclohexasiloxane | Organic compound | 0.14±0.01 | - | - | - | 0.10±0.03 | - | - | - | - | - |
| 39 | Ethanamine | Organic compound | - | - | 0.07±0.02 | - | 0.06±0.02 | - | - | - | - | - |
| 40 | Ethanol | Alcohol | - | - | - | - | - | - | - | - | - | 0.02±0.01 |
| 41 | Ethane | Hydrocarbon | - | - | - | - | - | - | - | - | 0.22±0.02 | - |
| 42 | Furan | Organic compound | - | - | 0.04±0.01 | - | - | - | - | - | - | - |
| 43 | Gulose | Carbohydrate | 0.23±0.07 | 0.15±0.01 | - | - | 0.33±0.01 | 0.12±0.03 | - | 0.14±0.05 | - | - |
| 44 | Glucopyranose | Carbohydrate | 0.77±0.07 | 0.45±0.02 | 0.97±0.08 | 0.19±0.06 | 0.29±0.01 | 0.18±0.05 | - | 3.75±0.20 | 1.51±0.11 | - |
| 45 | Glycine | Amino acid | 13.71±0.22 | 7.88±0.47 | 7.91±0.38 | 8.40±0.28 | 10.61±0.85 | - | 20.94±1.51 | 14.17±1.99 | 16.69±1.69 | 16.86±0.34 |
| 46 | Heptasiloxane | Siloxane | - | 1.21±0.73 | - | - | - | - | - | - | - | - |
| 47 | Hexadecanoic acid | Fatty acid | 0.59±0.01 | 1.28±0.04 | - | 0.10±0.04 | 0.28±0.14 | - | - | - | - | - |
| 48 | Hexanedioic acid | Organic compound | - | 3.17±1.91 | - | - | - | - | - | - | - | - |
| 49 | Hexamethyldisiloxane | Organic compound | 0.06±0.02 | - | 0.08±0.03 | - | - | 2.72±0.75 | - | 1.89±0.25 | - | - |
| 50 | Isopropyl Myristate | Organic compound | - | - | - | 1.95±0.28 | - | - | - | - | - | - |
| 51 | l-alanine | Amino acid | - | - | 0.35±0.02 | - | - | 2.32±0.84 | 3.18±0.92 | 1.98±0.17 | 2.52±0.59 | 1.75±0.24 |
| 52 | l-aspartic acid | Amino acid | - | 0.62±0.20 | 0.17±0.06 | - | - | 3.57±0.98 | - | 2.23±0.72 | - | - |
| 53 | L-Glutamine | Amino acid | 0.79±0.15 | 1.82±0.01 | 0.90±0.04 | 0.38±0.01 | 0.84±0.04 | 6.43±1.76 | - | 3.49±1.12 | 1.63±0.56 | - |
| 54 | L-isoleucine | Amino acid | 0.17±0.05 | 0.25±0.01 | 0.16±0.06 | 0.08±0.03 | 0.19±0.07 | 1.07±0.29 | 0.50±0.07 | 0.52±0.19 | - | 0.36±0.12 |
| 55 | l-leucine | Amino acid | 0.16±0.03 | 0.70±0.02 | 0.41±0.07 | 0.09±0.03 | 0.24±0.08 | 0.12±0.05 | 0.58±0.10 | 1.32±0.30 | 0.45±0.24- | 0.25±0.03 |
| 56 | l-lysine | Amino acid | - | - | - | - | - | 1.70±0.47 | - | 0.93±0.30 | - | - |
| 57 | l-mannopyranose | Carbohydrate | 0.07±0.02 | 0.19±0.01 | 0.10±0.01 | - | 0.04±0.01 | 0.05±0.01 | 0.24±0.06 | 0.15±0.05 | 0.65±0.06 | - |
| 58 | L-methionine | Amino acid |  |  |  |  |  | 0.12±0.03 | - | - | - | - |
| 59 | l-norvaline | Amino acid | - | - | 0.05±0.02 | - | 0.04±0.02 | - | - | - | 0.11±0.04 | - |
| 60 | l-proline | Amino acid | - | - | - | - | 0.51±0.16 | - | - | - | - | - |
| 61 | L-Serine | Amino acid | 0.34±0.03 | 0.40±0.02 | 0.29±0.01 | 0.18±0.06 | 0.37±0.01 | 1.37±0.37 | 0.84±0.10 | 0.78±0.23 | 0.70±0.24 | 0.34±0.03 |
| 62 | l-threonine | Amino acid | 0.35±0.01 | 0.87±0.02 | 0.45±0.16 | 0.16±0.05 | 0.34±0.04 | 0.92±0.37 | 0.97±0.13 | 0.98±0.19 | 0.79±0.27 | 0.38±0.02 |
| 63 | L-Tyrosine | Amino acid | - | - | - | - | - | 0.56±0.15 | - | 0.27±0.09 | - | - |
| 64 | l-valine | Amino acid | - | 1.88±0.07 | - | - | - | 0.16±0.04 | - | 12.31±3.97 | 0.23±0.08 | 0.10±0.01 |
| 65 | Lyxose | Carbohydrate | 0.10±0.07 | - | 0.25±0.01 | - | - |  |  |  |  |  |
| 66 | Malic acid | Organic compound | 0.29±0.01 | 0.36±0.01 | 0.52±0.02 | 0.23±0.08 | 0.32±0.01 | 0.45±0.02 | 0.37±0.14 | 0.46±0.15 | - | - |
| 67 | N-α-Acetyl-L-Lysine | Amino acid | - | 1.66±0.61 | 0.74±0.11 | - | 0.40±0.15 | - | - | - | - | - |
| 68 | Octadecanoic acid | Fatty acid | - | 0.20±0.01 | - | - | - | - | - | - | - | - |
| 69 | Oleic acid | Fatty acid | - | 1.49±0.48 | - | - | - | - | - | - | - | - |
| 70 | Picolinic acid | Organic compound | - | - | - | 0.05±0.02 | - | - | - | - | - | - |
| 71 | Pipecolic acid | Organic compound | - | - | - | - | - | 1.42±0.87 | - | - | - | - |
| 72 | Pentanamide | Monocarboxylic acid amide | - | - | - | - | 0.07±0.02 | - | - | - | - | - |
| 73 | Pentasiloxane | Siloxane | 0.16±0.01 | - | - | - | - | - | - | - | - | - |
| 74 | Phosphoric acid | Organic compound | 0.13±0.04 | 0.24±0.01 | - | - | - | 0.29± | - | - | - | - |
| 75 | Pentanedioic acid | Organic compound | 1.10±0.03 | 1.11±0.50 | 1.55±0.72 | 1.32±0.45 | 1.00±0.03 | 1.17±0.01 | 0.99±0.18 | 0.73±0.04 | 0.80±0.03 | 0.58±0.04 |
| 76 | Propane | Alkane | - | - | - | - | - | - | - | - | 4.01±0.41 | - |
| 77 | Propanedioic acid | Organic compound | - | - | - | - | - | 0.12±0.05 | - | - | - | 0.21±0.01 |
| 78 | Propanoic acid | Fatty acid | - | 0.18±0.08 | 0.15±0.08 | - | 0.03±0.01 | 0.18±0.05 | - | - | - | - |
| 79 | Pyridine | Organic compound | - | - | - | 0.15±0.01 | - | 0.15±0.04 | - | - | - | - |
| 80 | Pyrimidine | Organic compound | 0.08±0.03 | - | - | 0.05±0.02 | 0.09±0.03 | - | - | - | - | - |
| 81 | Pyroglutamic acid | Amino acid | 0.38±0.01 | 0.20±0.01 | - | - | - | - | 1.20±0.53 | - | - | - |
| 82 | Silanamine | Organic compound | 0.25±0.08 | 0.52±0.12 | 0.56±0.29 | 0.23±0.08 | 0.34±0.03 | 0.77±0.09 | 0.70±0.09 | 0.82±0.26 | 0.54±0.24 | 0.44±0.06 |
| 83 | Silanol, trimethyl-, phosphate (3:1) | Inorganic compound | 5.07±0.59 | 4.45±0.16 | 9.51±0.60 | 5.90±0.20 | 5.35±0.01 | 9.62±0.55 | 10.84±2.24 | 10.35±2.68 | 7.31±0.38 | 6.42±0.14 |
| 84 | Talose | Carbohydrate | 0.15±0.05 | - | 0.15±0.06 | - | - | - | - | 0.18±0.06 | - | - |
| 85 | Tetrasiloxane | Siloxane | - | - | - | - | 0.10±0.03 | - | - | - | - | - |
| 86 | Thymol-α-d-glucopyranoside | Carbohydrate | 7.18±1.96 | 8.74±1.74 | 7.89±1.93 | 4.21±1.43 | 10.89±1.69 | 5.62±0.64 | 8.32±0.36 | 5.36±0.28 | - | 7.88±0.01 |
| 87 | Tranexamic Acid | Organic compound |  | 0.06±0.01 |  | 0.01±0.01 | 0.04±0.01 | - | - | - | - | - |
| 88 | Trans-13-Octadecenoic acid | Fatty acid | - | 1.36±0.80 | - | - | - | - | - | - | - | - |
| 89 | Trisiloxane | Siloxane | 0.16±0.01 | 0.13±0.08 | 0.12±0.04 | 0.10±0.08 | 0.14±0.02 | - | - | - | - | - |
| 90 | Tris(trimethylsiloxy)ethylene | Hydrocarbon |  |  |  |  |  | - | 0.08±0.02 | - | - | - |
| 91 | Uracil | Pyrimidine | - | 0.06±0.04 | 0.07±0.03 | - | 0.08±0.03 | - | - | - | - | - |
