# Supplementary material for: Oleic acid as potential immunostimulant in metabolism pathways of hybrid grouper fingerlings (Epinephelus fuscoguttatus × Epinephelus lanceolatus) infected with Vibrio vulnificus
Source: Sci Rep. 2023 Aug 8;13:12830. doi: 10.1038/s41598-023-40096-7 (PMC10409752; doi:10.1038/s41598-023-40096-7)
Supplement: Supplementary file 3 — Supplementary Information 3. [file 41598_2023_40096_MOESM3_ESM.docx]

Supplementary Table 2 Pathway analysis based on the metabolites present in the liver samples from the survived-infected grouper.

|  | **Metabolic pathway** | **p value** | **–log 10 (p)** | **FDR** | **impact value** |
| --- | --- | --- | --- | --- | --- |
| 1 | Aminoacyl-tRNA biosynthesis* | 8.77×10^-7^ | 6.06 | 7.37×10^-5^ | 0.17 |
| 2 | Valine, leucine, and isoleucine biosynthesis* | 5.37×10^-5^ | 4.27 | 2.00×10^-3^ | 0.00 |
| 3 | Alanine, aspartate, and glutamate metabolism* | 0.01 | 2.06 | 0.24 | 0.35 |
| 4 | Biosynthesis of unsaturated fatty acids* | 0.02 | 1.67 | 0.45 | 0.00 |
| 5 | Arginine biosynthesis | 0.07 | 1.16 | 0.89 | 0.00 |
| 6 | Glyoxylate and dicarboxylate metabolism | 0.08 | 1.12 | 0.89 | 0.15 |
| 7 | Glycine, serine, and threonine metabolism | 0.08 | 1.09 | 0.89 | 0.49 |
| 8 | beta-Alanine metabolism | 0.11 | 0.97 | 0.89 | 0.00 |
| 9 | Pantothenate and CoA biosynthesis | 0.11 | 0.97 | 0.89 | 0.00 |
| 10 | Pentose and glucuronate interconversions | 0.12 | 0.93 | 0.89 | 0.07 |
| 11 | Linoleic acid metabolism | 0.12 | 0.92 | 0.89 | 1.00 |
| 12 | Valine, leucine, and isoleucine degradation | 0.13 | 0.90 | 0.89 | 0.00 |
| 13 | Propanoate metabolism | 0.16 | 0.80 | 0.98 | 0.00 |
| 14 | D-Glutamine and D-glutamate metabolism | 0.17 | 0.80 | 0.98 | 0.00 |
| 15 | Nitrogen metabolism | 0.17 | 0.80 | 0.98 | 0.00 |
| 16 | Arginine and proline metabolism | 0.34 | 0.47 | 1.00 | 0.08 |
| 17 | Amino sugar and nucleotide sugar metabolism | 0.35 | 0.46 | 1.00 | 0.00 |
| 18 | Nicotinate and nicotinamide metabolism | 0.36 | 0.44 | 1.00 | 0.00 |
| 19 | Pyrimidine metabolism | 0.37 | 0.43 | 1.00 | 0.09 |
| 20 | Histidine metabolism | 0.38 | 0.42 | 1.00 | 0.00 |
| 21 | Butanoate metabolism | 0.38 | 0.42 | 1.00 | 0.00 |
| 22 | Selenocompound metabolism | 0.44 | 0.36 | 1.00 | 0.00 |
| 23 | Citrate cycle (TCA cycle) | 0.47 | 0.33 | 1.00 | 0.03 |
| 24 | Sphingolipid metabolism | 0.49 | 0.31 | 1.00 | 0.00 |
| 25 | Pentose phosphate pathway | 0.51 | 0.30 | 1.00 | 0.00 |
| 26 | Glycolysis / Gluconeogenesis | 0.57 | 0.25 | 1.00 | 0.00 |
| 27 | Galactose metabolism | 0.58 | 0.24 | 1.00 | 0.05 |
| 28 | Glutathione metabolism | 0.59 | 0.23 | 1.00 | 0.09 |
| 29 | Cysteine and methionine metabolism | 0.65 | 0.18 | 1.00 | 0.02 |
| 30 | Fatty acid degradation | 0.71 | 0.15 | 1.00 | 0.00 |
| 31 | Fatty acid elongation | 0.72 | 0.15 | 1.00 | 0.00 |
| 32 | Fatty acid biosynthesis | 0.78 | 0.11 | 1.00 | 0.02 |
| 33 | Purine metabolism | 0.88 | 0.05 | 1.00 | 0.00 |

*Significant difference p< 0.05
